# New Chiral Ebselen Analogues with Antioxidant and Cytotoxic Potential

**DOI:** 10.3390/molecules22030492

**Published:** 2017-03-20

**Authors:** Agata J. Pacuła, Katarzyna B. Kaczor, Jędrzej Antosiewicz, Anna Janecka, Angelika Długosz, Tomasz Janecki, Andrzej Wojtczak, Jacek Ścianowski

**Affiliations:** 1 Department of Organic Chemistry, Faculty of Chemistry, Nicolaus Copernicus University, Gagarina 7, 87-100 Torun, Poland; pacula@doktorant.umk.pl (A.J.P.); awojt@umk.pl (A.W.); 2 Department of Bioenergetics and Physiology of Exercise, Medical University of Gdansk, Debinki 1, 80-211 Gdansk, Poland; katarzyna.kaczor@gumed.edu.pl (K.B.K.); jant@gumed.edu.pl (J.A.); 3 Department of Biomolecular Chemistry, Faculty of Medicine, Medical University of Lodz, Mazowiecka 6/8, 92-215 Lodz, Poland; anna.janecka@umed.lodz.pl (A.J.); angelika.dlugosz@stud.umed.lodz.pl (A.D.); 4 Institute of Organic Chemistry, Lodz University of Technology, Zeromskiego 116, 90-924 Lodz, Poland; tomasz.janecki@p.lodz.pl; 5 Department of Biochemistry, Gdansk University of Physical Education and Sport, Kazimierza Gorskiego 1, 80-336 Gdansk, Poland

**Keywords:** organoselenium compounds, terpenes, antioxidant activity, biological activity

## Abstract

New chiral camphane-derived benzisoselenazol-3(2*H*)-ones and corresponding diselenides have been synthetized using a convenient one-pot procedure. Se-N bond was efficiently converted to an Se-Se bond, which could also be easily re-oxidized to the initial benzisoselenazolone moiety. The antioxidant activity of camphor derivatives was evaluated and compared to the reactivity of a series of *N*-amino acid benzisoselenazol-3(2*H*)-ones obtained by a modified procedure involving the improved synthesis and isolation of the diseleno bis(dibenzoic) acid. The most efficient peroxide scavengers, *N*-bornyl and *N*-leucine methyl ester benzisoselenazol-3(2*H*)-ones, were further evaluated as cytotoxic agents on four cancer cell lines (MCF-7, HEP G2, HL 6, and DU 145) and normal cell line PNT1A. The highest antiproliferative potential was evaluated for two compounds bearing a 3-methylbutyl carbon chain, *N*-leucine methyl ester and *N*-3-methylbutyl benzisoselenazol-3(2*H*)-ones.

## 1. Introduction

Since it was proven that selenium is an essential micronutrient mainly associated with the antioxidant defense system of the living cell, the discovery of ebselen (*N*-phenylbenzisoselenazol-3(2*H*)-one) **1** has been considered a milestone in this constantly developing field of research [1]. This organoselenium compound is able to act as selenocysteine (Sec), thus mimicking the activity of glutathione peroxidase (GPx). Sec is the key element of the enzymes active site that provides GPx with the unique ability to reduce hydrogen peroxide and other reactive oxygen and nitrogen species [2]. This unique property makes ebselen a potent bioactive molecule which is effective in the treatment of various diseases including diabetes [3,4,5,6], Alzheimer’s disease [7], stroke [8], hearing loss [9], and cancer [10]. It is also non-toxic, as the selenium atom incorporated in its structure is not bioavailable [11].

**Scheme 1:**
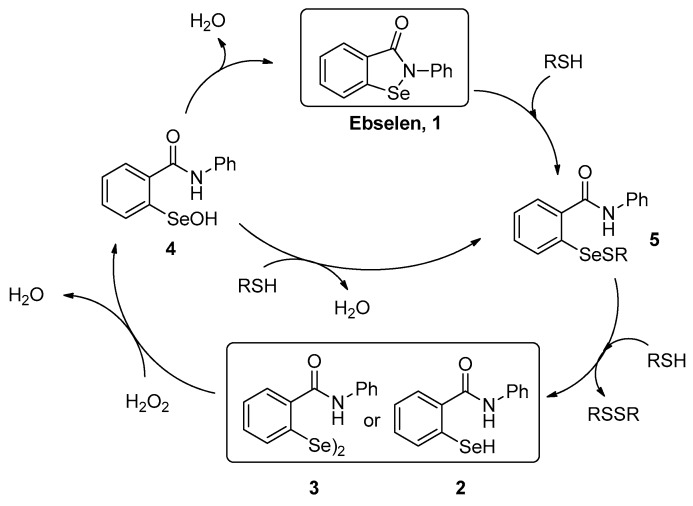
Probable catalytic pathways for the reduction of H_2_O_2_ by ebselen.

The mechanism of peroxides reduction by ebselen **1** is controversial and several activity pathways have been proposed. It was postulated that the elimination of peroxide is performed by a specific active species, formed from ebselen **1**. According to different assays, **1** can be transformed into selenol **2** [12] or diselenide **3** [13], which can reduce hydrogen peroxide forming selenic acid **4**. Elimination of water from acid **4** regenerates ebselen **1** (Scheme 1).

In both cases, the rate determining the step of the cycle is the cleavage of the Se-S bond, in the selenenylsulfide **5** and the formation of the active organoselenium species **2** or **3**. Our recent studies confirmed this assumption, showing that ebselen derivatives undergo facile Se-N bond cleavage and rapidly form the corresponding diselenides, exhibiting high antioxidant potential [14]. To further examine this issue, we synthesized a series of new chiral ebselen derivatives, transformed them to corresponding diselenides, and compared their antioxidant activity. It seems probable that the direct application of a diselenide could increase the rate of peroxide reduction.

**Scheme 2:**
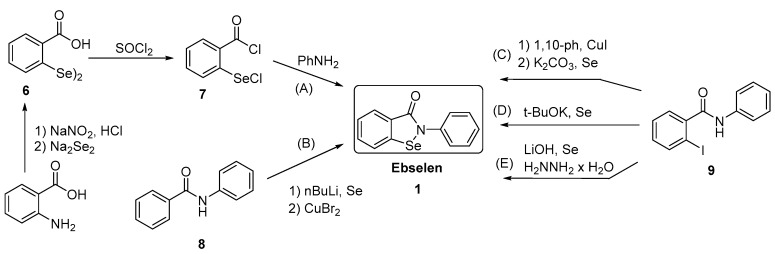
Methodologies to synthesize ebselen **1**.

So far, the synthesis of *N*-phenylbenzisoselenazol-3(2*H*)-one **1** has been accomplished by a multi-step synthesis based on the formation of 2,2-diselenobis(benzoic acid) **6**, followed by the conversion to corresponding dichloride **7** with thionyl chloride and further reaction with aniline (Method A) [15] or *o*-metalation of benzamide **8**, selenium insertion, and oxidative cyclization promoted by copper bromide (Method B) [16]. Several methods in which *N*-phenyl-*o*-iodobenzamide **9** is used as a starting material are described. Ebselen **1** was obtained by treatment with a copper catalyst and selenium in the presence of potassium carbonate as a base (Method C) [17], a nucleophilic reagent formed from selenium and potassium *tert*-butoxide (Method D) [18] or by the methodology presented in our previous paper, in which lithium diselenide, formed in the reaction of lithium hydroxide and elemental selenium in the presence of hydrazine hydrate, is applied (Method E) [19] (Scheme 2).

Although various benzisoselenazolones have been synthetized and tested as bio-molecules, there are only few examples of chiral ebselen derivatives [18,20,21,22]. The aim of our research was to fill this gap and combine the benzisoselenazolone structure with a chiral terpene scaffold. Terpenes possess several significant properties. As natural products abundant in plants, they provide a broad spectrum of structurally diverse motifs and were used not only as fragrance or taste carriers but also as pharmacologically potent agents [23]. In our research group, we efficiently used terpenes to obtain a broad spectrum of various chiral organoselenium derivatives including selenols, selenides, and diselenides [24,25,26,27,28,29,30,31].

Herein, we report the synthesis of benzisoselenazolones and corresponding diselenides substituted on the nitrogen atom with monoterpene skeletons from camphane group. We also tested the obtained compounds for their antioxidant and anticancer activity. Obtained results were additionally compared to the activity of a series of other chiral, naturally derived benzisoselenazolones bearing amino acid substituents.

## 2. Results and Discussion

**Scheme 3:**
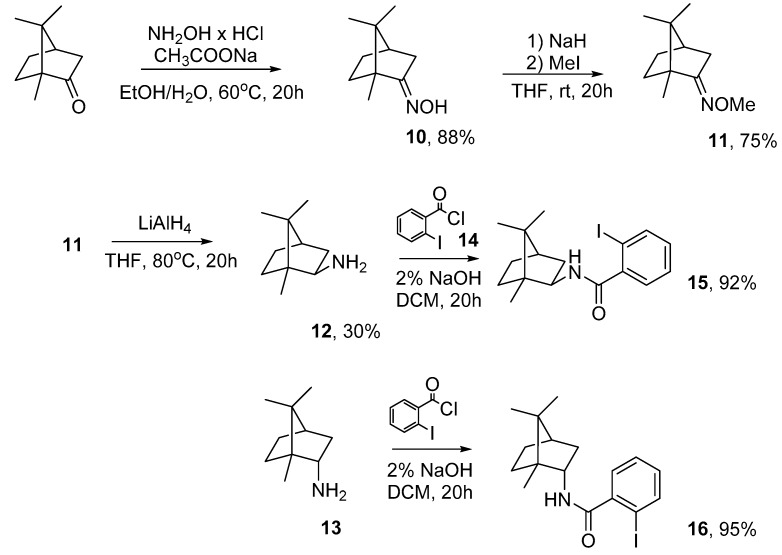
Synthesis of *N*-isobornyl and *N*-bornyl-*o*-iodobenzamides **15** and **16**.

In the conducted research, we planned to use and compare the efficiency of two metal-free procedures (method D and E) in the synthesis of new camphane-derived benzisoselenazolones and corresponding diselenides. The first step included the synthesis of *N*-terpenyl-*o*-iodobenzamides **15** and **16** used as substrates. *R*(−)-Isobornylamine **12** was obtained from camphor by a three-step procedure consisting of formation of corresponding oxime **10**, its conversion to oxime methyl ether **11**, and reduction of **11** with lithium aluminium hydride to amine **12**. During the reduction of oxime methyl ether, we observed the formation of isobornyl amine **12** and bornyl amine **13** (64:36). Isobornyl amine **12** was separated by column chromatography (silica gel, dichloromethane). Iodobenzamides **15** and **16** were obtained from the reaction of acid chloride **14** with previously synthetized amine **12** and commercially available *R*(+)-bornylamnie **13** (Scheme 3).

**Scheme 4:**
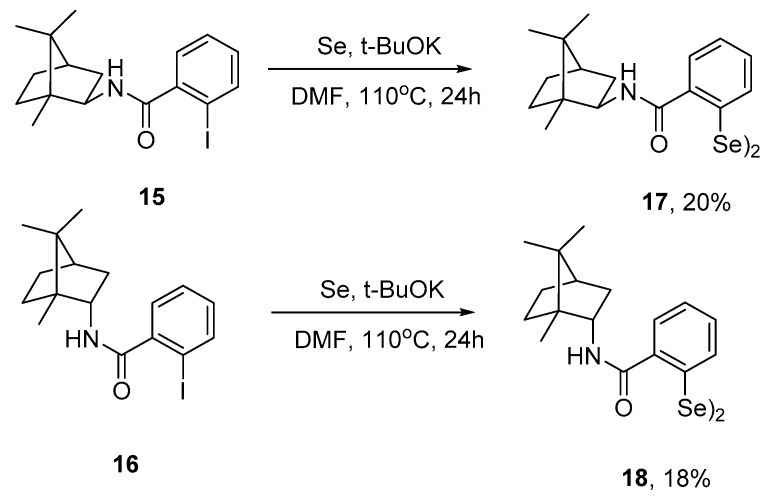
Synthesis of diselenides **17** and **18**.

In the next step, we decided to obtain benzisoselenazolones by the reaction of amides **15** and **16** with nucleophilic reagent formed in situ from elemental selenium and potassium *tert*-butoxide (Method D). Unfortunately, corresponding diselenides **17** and **18** were obtained in low yields (Scheme 4).

**Scheme 5:**
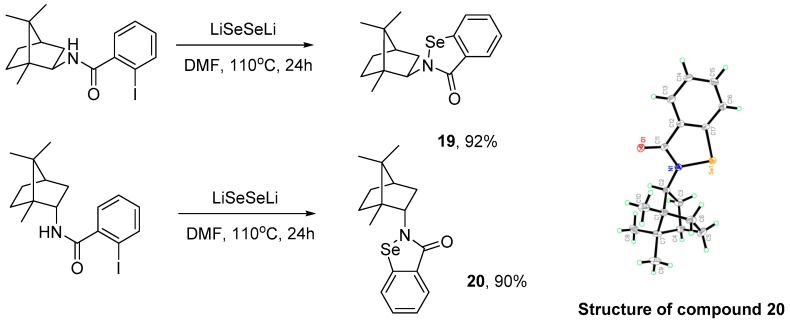
Synthesis of *N*-isobornyl and *N*-bornyl benzisoselenazol-3(2*H*)-ones **19** and **20**.

These results prompted us to modify the procedure and design a new methodology in which the nucleophile was changed to lithium diselenide, formed from lithium hydroxide and elemental selenium in the presence of hydrazine hydrate. To our delight, this modification gave expected benzisoselenazolones **19** and **20** in high yields. The structure of compound **20** was additionally confirmed by an X-ray analysis (Scheme 5).

The absolute configuration of compound **20**, determined by the Flack method [32] is (1*R*,2*S*,4*R*). The bulk of the *N*-bornyl moiety seems to be the largest among the chiral benzisoselenazolones reported so far [13,15]. Two bonds formed by Se1 are almost identical, with the Se1-N1 and Se1-C17 distances being 1.883(2) and 1.887(2) Å, respectively and the N1-Se1-C17 angle is 85.89(10) deg. These values are similar to those found for other chiral derivatives. However, a search in the Cambridge Structural Database [33] revealed that N-Se-C angle values range from 77.05 to 90.39 deg, with the smaller angles for compounds having the additional substituent at Se atom. Comparison revealed that Se-N and Se-C bond distances reported here were significantly longer than those Se-C 1.868(4) and Se-N 1.870(5) Å reported for the analog containing two ebselen moieties linked by ethylene bridge [34]. In that structure, the C-Se-N angle of 84.97(18) is significantly smaller than that in the structure reported here. In selenoxide, analog of ebselen [35], the Se-N and Se-C bonds of 1.899(3) and 1.928(3) Å, respectively, are longer than those found in compound **20** reported here. Also, the N-Se-C angle 84.68(13) deg was significantly smaller than that in **20**. In the structure of ebselen with no substituent at N [36], the significant difference up to 0.039 Å was reported for Se-N and Se-C distances, and the C-Se-N angle was significantly smaller than in the structure reported here. However, geometry of N-Se-C fragment in compound containing *N*-Met [37] was almost identical with that reported here. That might result from the steric and electronic effect of Se=O bond in selenoxide. Contrary to this, the values of Se-C 1.856(2), Se1-N 1.916(2) and C-Se-N 84.33(10) reported for of ebselen [38] differed significantly from those in the structure reported here. The comparison given above indicates that the electronic effect and not only the bulk or nature of *N*-substituent affect the ebselen geometry in a significant manner. Geometry of the bornyl moiety is typical for such systems. Conformation of the molecule is described with Se1-N1-C2-C1 and Se1-N1-C2-C3 torsion angles being 93.9(2) and −27.7(3) deg, respectively. See detailed information about NMR spectra and crystallographic data in Supplementary Materials.

**Scheme 6:**
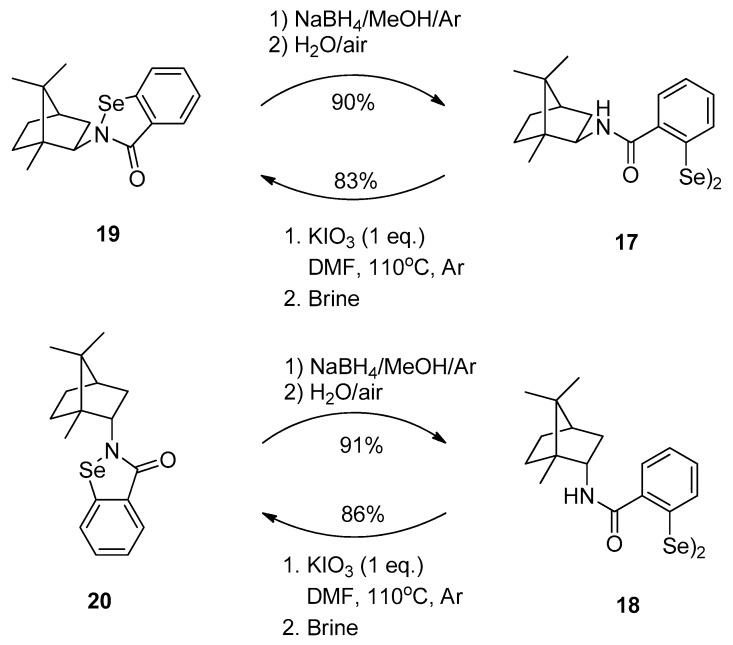
Cleavage and regeneration of Se-N bond.

Benzisoselenazolones **19** and **20** were further quantitatively converted to corresponding diselenides **17** and **18** using sodium borohydride. On the other hand, the Se-N bond could be efficiently regenerated in the reaction with potassium iodate used as an oxidant (Scheme 6).

To compare the biological activity of the synthetized compounds to other chiral ebselen analogues, a series of amino acid derivatives was obtained and similarly evaluated. To achieve this goal, several amino acid methyl esters were converted into *N*-substituted-*o*-iodobenzamides. Conversion to the final organoselenium derivatives using the methodology developed in our research group (Method E) was unsuccessful due to the hydrolysis of the ester moiety. As procedures involving cyclization of iodobenzamides using nucleophilic selenium reagents could not be used, we focused on the commonly used multistep Method A. During this investigation, we developed a revised version of this method, i.e., a convenient procedure to obtain the 2,2-diselenobis(benzoic acid) **6**. Synthesis of **6** significantly limits the overall yield of the reaction, thus improving the efficiency of this step highly upgrades the methodology. Acid **6** is usually prepared from the diazonium salt of anthranilic acid followed by substitution with sodium diselenide. The yield of the reaction depends on the procedure by which sodium diselenide is formed and by the efficiency of the final product isolation. Sodium diselenide can be prepared using different reagents, elemental selenium and sodium hydroxide in the presence of hydrazine in methanol [39], with addition of rongalite in water [40] or from elemental selenium and sodium borohydride in water, with or without NaOH [41,42]. We turned our attention to the last procedure, in which sodium diselenide is synthesized from Se and NaBH_4_ in NaOH_aq_. By changing the reaction conditions, i.e., lower temperature and longer reaction time, we were able to obtain pure Na_2_Se_2_, eliminating the common formation of Na_2_Se. Next, we optimized the purification procedure of the final product. Synthesized 2,2-diselenobis(benzoic acid) **6** is often contaminated with salicylic acid which is hard to separate. Multiple rinsing of the crude product with boiling water enabled to obtain the final product as pure solid in 90% yield.

**Scheme 7:**
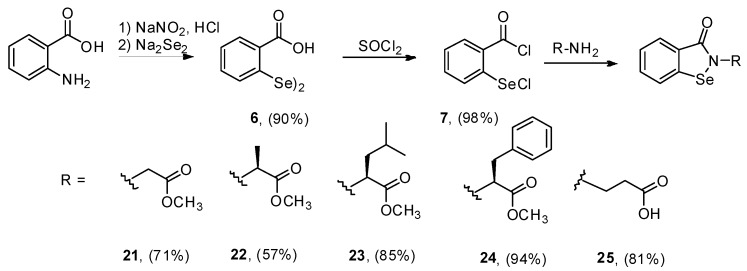
Synthesis of amino acid derivatives **21**–**25**.

The revised procedure (Method A) was used to synthesize *N*-substituted benzisoselenazol-3(2*H*)-ones derived from glycine **21**, *L*-alanine **22**, *L*-leucine **23**, *L*-phenylalanine **24** and β-alanine **25** [22] (Scheme 7).

All obtained terpene and amino acid derivatives were tested as antioxidants using NMR activity assay proposed by Iwaoka and co-workers. The ability to eliminate hydrogen peroxide was equal to the rate of the oxidation of dithiol to disulfide. Formation of the product was observed by ^1^H-NMR spectra performed in selected time intervals [43]. The results are presented in Table 1.

The highest reactivity was found for *N*-bornyl **20** and *N*-leucine methyl ester **23** benzisoselenazol-3(2*H*)-ones. Total conversion of DTT^red^ to DTT^ox^ (DDT-Dithiotreitol) was observed after 5 and 3 min, respectively. Other terpene derivatives **17**–**19** exhibited much lower antioxidant potential, and no antioxidant potential was observed for other amino acid benzisoselenazolones. This result led us to the conclusion that the structure of leucine, bearing a 2-methylpropyl chain on the α-carbon, could be the reason of this high reactivity. Thus, for further anticancer activity assays, we chose to evaluate the cytotoxic potential of compounds **20** and **23**, for which the best results were obtained in the NMR activity test.

Additionally, we decided to test the anticancer activity of *N*-3-methylbutyl benzisoselenazol-3(2*H*)-one **26** presented in our previous paper [19], bearing a carbon chain analogous to the one present in the structure of leucine. The cytotoxic activity was evaluated on four cancer cell lines: MCF-7 (breast carcinoma), HEP G2 (liver cancer), HL60 (human promyelocytic leukemia), DU 145 (prostate cancer), and non-cancerous cell line PNT1A. The results are collected in Table 2.

The highest antiproliferative activity was observed for *N*-leucine methyl ester derivative **23** against MCF-7, HEP G2, and HL60 cells. This compound also exhibited the best antioxidant potential. Its activity against MCF-7, HEP G2, and HL60 was relatively good and was compared with the data obtained for carboplatin. Structurally similar *N*-3-methylbutyl benzisoselenazol-3(2*H*)-one **26** was also active against all three cancer cell lines and had low cytotoxicity in HEP G2 cell line, on the contrary to data obtained on chiral *N*-bornyl derivative **20**, revealing that the presence of α-carbon 2-methylpropyl chain could be a structural motif providing the bio-activity.

## 3. Experimental

### 3.1. General

^1^H-NMR spectra were obtained at 400 or 700 MHz and chemical shifts were recorded relative to SiMe_4_ (δ 0.00) or solvent resonance (CDCl_3_ δ 7.26, CD_3_OD δ 3.31). Multiplicities were given as s (singlet), d (doublet), dd (double doublet), ddd (double double doublet), t (triplet), td (triple doublet), dt (double triplet), and m (multiplet). The number of protons (n) for a given resonance was indicated by *n*H. Coupling constants were reported as a *J* value in Hz. ^13^C-NMR spectra were acquired at 100.6 MHz and chemical shifts were recorded relative to solvent resonance (CDCl_3_ δ 77.25). NMR spectra were carried out using ACD/NMR Processor Academic Edition (Advanced Chemistry Development, Toronto, ON, Canada). Original NMR spectra and crystallographic data are included in SM. Commercially available solvents DMF (*N*,*N*-dimethylformamide), DCM (dichloromethane), and MeOH (Sigma Aldrich, St. Louis, MO, USA), and chemicals were used without further purification. Column chromatography was performed using Merck 40-63D 60 Å silica gel (Merck, Darmstadt, Germany).

### 3.2. Procedures and Analysis Data

#### 3.2.1. (1*R*)-Camphor Oxime (**10**)

To a solution of hydroxylamine hydrochloride (0.164 mol) and sodium acetate (0.197 mol) in water (100 mL), (+)-camphor (0.131 mol) dissolved in ethanol (65 mL) was added. The mixture was stirred at 60 °C for 20 h. Ethanol was evaporated and the precipitated oxime filtrated under reduce pressure. The product was used without further purification [44].

Yield: 88%, ^1^H-NMR (400 MHz, CDCl_3_) δ = 0.82 (s, 3H, CH_3_), 0.94 (s, 3H, CH_3_), 1.02 (s, 3H, CH_3_), 1.20–1.30 (m, 1H), 1.43–1.52 (m, 1H), 1.72 (td, *J* = 4.0, 12.4 Hz, 1H), 1.80–1.91 (m, 1H), 1.93 (t, *J* = 4, 4 Hz, 1H), 2.07 (d, *J* = 18.0 Hz, 1H), 2.57 (dt, *J* = 4.4, 18.0 Hz, 1H) ppm.

#### 3.2.2. (1*R*)-Camphor *O*-methyloxime (**11**)

To a suspension of sodium hydride (0.183 mol) in anhydrous petroleum ether a solution of (1*R*)-Camphor oxime 10 (0.093 mol) in anhydrous THF (90 mL) was added portionwise. The mixture was stirred for 0.5 h at room temperature and methyl iodide (0.111 mol) was added. The mixture was stirred for additional 20 h. THF was evaporated, and the formed precipitate was washed with water (50 mL) and extracted with petroleum ether (2 × 50 mL). Combined organic layers were dried over anhydrous magnesium sulphate and evaporated. The product was used without further purification [45].

Yield: 75%, ^1^H-NMR (700 MHz, CDCl_3_) δ = 0.82 (s, 3H, CH_3_), 0.94 (s, 3H, CH_3_), 1.04 (s, 3H, CH_3_), 1.20–1.30 (m, 1H), 1.43–1.52 (m, 1H), 1.72 (td, *J* = 4.0, 12.4 Hz, 1H), 1.82–1.88 (m, 1H), 1.90 (t, *J* = 4.2 Hz, 1H), 2.07 (d, *J* = 18.2 Hz, 1H), 2.49 (dt, *J* = 7.7, 17.5 Hz, 1H), 3.85 (s, OCH_3_, 3H) ppm.

#### 3.2.3. (*R*)-(−)-Isobornylamine (**12**)

To a suspension of LiAlH_4_ (0.066 mol) in anhydrous THF (50 mL) a solution of (1*R*)-camphor *O*-methyloxime **11** (0.33 mol) in THF (25 mL) was slowly added. The mixture was stirred under reflux for 24 h. After cooling to room temperature H_2_O (2 mL), 10% NaOH (3 mL) and H_2_O (6 mL) were added dropwise. The formed suspension was extracted with diethyl ether. Combined organic layers were dried and evaporated under reduce pressure. The crude product was isolated by column chromatography (silica gel, DCM).

Yield: 30%, ^1^H-NMR (700 MHz, CDCl_3_) δ = 0.84 (s, 3H, CH_3_), 0.89 (s, 3H, CH_3_), 0.99 (s, 3H, CH_3_), 1.00–1.05 (m, 1H), 1.08–1.12 (m, 1H), 1.52–1.59 (m, 4H), 1.67–1.72 (m, 2H), 1.77 (dd, *J* = 8.4, 12.6 Hz, 1H), 2.74 (dd, *J* = 4.9, 9.1 Hz, 1H) ppm.

#### 3.2.4. General Procedure for the Synthesis of Compounds **15** and **16**

To a solution of an amine (1.0 mmol) in DCM (2 mL), 4.4 mL of 2% NaOH was added. The mixture was cooled to 0 °C and *o*-iodobenzoic acid chloride (1.1 mmol) dissolved in DCM (3 mL) was added dropwise. After stirring at room temperature for 20 h, the product was extracted with DCM, and combined organic layers were washed with 10% NaHCO_3_ and dried over magnesium sulphate. The solvent was removed under reduce pressure to obtain the product as white solid.

*N-Isobornyl-o-iodobenzamide* (**15**). Yield: 92%, ${\left[\alpha \right]}_{D}^{25}$ = −73.0 (*c* 1, CHCl_3_), ^1^H-NMR (400 MHz, CDCl_3_) δ = 0.83 (s, 3H, CH_3_), 0.90 (s, 3H, CH_3_), 0.95 (s, 3H, CH_3_), 1.14–1.22 (m, 1H), 1.31–1.38 (m, 1H), 1.55–1.65 (m, 1H), 1.65–1.77 (m, 3H), 1.93 (dd, *J* = 8.4, 12.0 Hz, 1H), 4.07 (dt, *J* = 4.8, 8.8 Hz, 1H), 5.75 (d, *J* = 8.0 Hz, 1H, NH), 7.01–7.09 (m, 1H_ar_), 7.30–7.35 (m, 2H_ar_), 7.80 (d, *J* = 7.6 Hz, 1H_ar_) ppm; ^13^C-NMR (100.6 Hz, CDCl_3_) δ = 12.2 (CH_3_), 20.2 (CH_3_), 20.5 (CH_3_), 27.0 (CH_2_), 36.0 (CH_2_), 38.9 (CH_2_), 44.9 (CH), 47.2 (C), 48.8 (C), 57.4 (CH), 92.3 (C_ar_), 128.2 (CH_ar_), 128.3 (CH_ar_), 130.9 (CH_ar_), 139.9 (CH_ar_), 142.6 (C_ar_), 168.7 (C=O) ppm; Elemental Anal. Calcd for C_17_H_22_INO (383.267): C, 53.27; H, 5.79. Found: C, 53.08; H, 5.81.

*N-Bornyl-o-iodobenzamide* (**16**). Yield: 95%, m.p. 119–121 °C, ${\left[\alpha \right]}_{D}^{25}$ = +21.5 (*c* 1, CHCl_3_), ^1^H-NMR (700 MHz, CDCl_3_) δ = 0.90 (s, 3H, CH_3_), 0.97 (s, 3H, CH_3_), 1.00 (s, 3H, CH_3_), 1.17–1.22 (m, 1H), 1.42–1.47 (m, 1H), 1.54–1.60 (m, 3H), 1.71 (t, *J* = 4.2 Hz, 1H), 1.78–1.83 (m, 1H), 4.42–4.46 (m, 1H), 5.75 (d, *J* = 7.0 Hz, 1H, NH), 7.10 (dt, *J* = 2.1, 7.7 Hz, 1H, 1H_ar_), 7.37–7.42 (m, 2H_ar_), 7.87 (dd, *J* = 0.7, 7.7 Hz, 1H_ar_) ppm; ^13^C-NMR (100.6 Hz, CDCl_3_) δ = 13.9 (CH_3_), 18.7 (CH_3_), 19.8 (CH_3_), 28.1 (CH_2_), 28.3 (CH_2_), 37.4 (CH_2_), 44.9 (CH), 48.3 (C), 49.6 (C), 54.6 (CH), 92.3 (C_ar_), 128.1 (CH_ar_), 128.4 (CH_ar_), 130.9 (CH_ar_), 139.8 (CH_ar_), 142.9 (C_ar_), 169.9 (C=O) ppm; Elemental Anal. Calcd for C_17_H_22_INO (383.267): C, 53.27; H, 5.79. Found: C, 53.13; H, 5.85.

#### 3.2.5. General Procedure for the Synthesis of Compounds **16** and **17**

To a solution of benzisoselenazolone **19** and **20** (1.0 mmol) in methanol (10 mL) cooled to 0 °C, sodium borohydride (1.0 mmol) was added and the mixture was stirred for 1 h. Water (15 mL) was added and the mixture was oxidized with air for 1 h. The formed precipitate was filtered and dried in air.

*2,2′-Diselenobis(N-isobornylbenzamide)* (**17**). Yield: 90%, m.p. 148–150 °C, ${\left[\alpha \right]}_{D}^{25}$ = −0.9 (*c* 1, CHCl_3_), ^1^H-NMR (400 MHz, CDCl_3_) δ = 0.89 (s, 6H, 2× CH_3_), 0.98 (s, 6H, 2× CH_3_), 1.04 (s, 6H, 2× CH_3_), 1.20–1.27 (m, 2H), 1.37–1.43 (m, 2H), 1.61–1.81 (m, 6H), 1.83 (t, *J* = 4.4 Hz, 2H), 2.01 (dd,* J* = 9.2, 13.2 Hz, 2H), 4.17 (dt, *J* = 5.2, 8.8 Hz, 2H), 6.14 (d, *J* = 8.8 Hz, 2H, 2× NH), 7.22 (dt, *J* = 1.2, 7.2 Hz, 2H_ar_), 7.28 (dt, *J* = 1.6, 7.2 Hz, 2H_ar_), 7.41 (dd, *J* = 1.6, 7.6 Hz, 2H_ar_), 7.91 (dd, *J* = 1.2, 8.0 Hz, 2H_ar_) ppm; ^13^C-NMR (100.6 Hz, CDCl_3_) δ = 11.8 (2× CH_3_), 20.2 (2× CH_3_), 20.3 (2× CH_3_), 27.0 (2× CH_2_), 35.8 (2× CH_2_), 39.0 (2× CH_2_), 44.9 (2× CH), 47.2 (2× C), 48.9 (2× C), 57.4 (2× CH), 126.0 (2× C_ar_), 126.1 (2× CH_ar_), 131.4 (2× CH_ar_), 131.6 (2× CH_ar_), 133.0 (2× C_ar_), 133.5 (2× C_ar_), 167.4 (2× C=O) ppm; ^77^Se (76.3 MHz, DMSO), δ = 450.58 ppm; Elemental Anal. Calcd for C_34_H_44_N_2_O_2_Se_2_ (670.645): C, 60.89; H, 6.61. Found: C, 60.52; H, 6.55.

*2,2′-Diselenobis(N-bornylbenzamide)* (**18**). Yield: 91%, m.p. 208–210 °C, ${\left[\alpha \right]}_{D}^{25}$ = −80.0 (*c* 1, CHCl_3_), ^1^H-NMR (700 MHz, CDCl_3_) δ = 0.94 (s, 6H, 2× CH_3_), 1.00 (s, 6H, 2× CH_3_), 1.09 (s, 6H, 2× CH_3_), 1.41–1.47 (m, 6H), 1.55–1.59 (m, 2H), 1.81 (t, *J* = 4.2 Hz, 2H), 1.92–1.95 (m, 2H), 2.00–2.05 (m, 2H), 2.49–2.55 (m, 2H), 5.06 (ddd, *J* = 2.1, 4.9, Hz, 2H, 2× NH), 7.44 (dt, *J* = 0.7, 7.0 Hz, 2H_ar_), 7.60 (dt, *J* = 1.4, 5.6 Hz, 2H_ar_), 7.62–7.65 (m, 2H_ar_), 8.06 (dd, *J* = 0.7, 7.7 Hz, 2H_ar_) ppm; ^13^C-NMR (100.6 Hz, CDCl_3_) δ = 13.8 (2× CH_3_), 18.6 (2× CH_3_), 19.8 (2× CH_3_), 28.2 (2× CH_2_), 28.4 (2× CH_2_), 37.8 (2× CH_2_), 44.9 (2× CH), 48.3 (2× C), 49.8 (2× C), 54.5 (2× CH), 126.0 (2× CH_ar_), 126.4 (2× CH_ar_), 131.4 (2× CH_ar_), 131.6 (2× CH_ar_), 132.8 (2× C_ar_), 133.8 (2× C_ar_), 168.3 (2× C=O) ppm; ^77^Se (76.3 MHz, DMSO), δ = 450.03 ppm; Elemental Anal. Calcd for C_34_H_44_N_2_O_2_Se_2_ (670.645): C, 60.89; H, 6.61. Found: C, 60.63; H, 6.64.

#### 3.2.6. General Procedure for the Synthesis of Compounds **19** and **20**

Selenium powder (1.2 mmol) and lithium hydroxide (3.6 mmol) were weighed into a single neck flask under argon atmosphere and dissolved in DMF (3 mL). Hydrazine hydride (8.0 mmol) was added dropwise, and the mixture was heated to 120 °C and stirred for 15 min. After cooling to room temperature, the amide (1.0 mmol) dissolved in DMF (2 mL) was added. The reaction mixture was heated to 120 °C and stirred for 20 h under argon atmosphere. The solution was cooled to room temperature and 25 mL of brine was added. The mixture was stirred for 20 h. The obtained precipitate was filtered under vacume, washed with water, and dried in air. The crude product was purified by column chromatography (silica gel, DCM:MeOH 0.8:99.2).

*N-Isobornyl-1,2-benzisoselenazol-3(2H)-one* (**19**). Yield: 92%, m.p. 205–207 °C, ${\left[\alpha \right]}_{D}^{25}$ = −59.0 (*c* 1, CHCl_3_), ^1^H-NMR (400 MHz, CDCl_3_) δ = 0.91 (s, 3H, CH_3_), 0.93 (s, 3H, CH_3_), 1.17 (s, 3H, CH_3_), 1.25–1.34 (m, 2H), 1.63–1.71 (m, 1H), 1.80–1.88 (m, 2H), 2.00–2.07 (m, 2H), 4.77 (t, *J* = 8.4 Hz, 1H), 7.42 (ddd, *J* = 2.0, 6.0, 8.0 Hz, 1H_ar_), 7.55–7.60 (m, 2H_ar_), 8.04 (d, *J* = 7.6 Hz, 1H_ar_) ppm; ^13^C-NMR (100.6 Hz, CDCl_3_) δ = 11.3 (CH_3_), 21.2 (CH_3_), 22.0 (CH_3_), 26.5 (CH_2_), 37.1 (CH_2_), 39.3 (CH_2_), 45.2 (CH), 46.7 (C), 51.4 (C), 63.3 (CH), 123.2 (CH_ar_), 126.0 (CH_ar_), 128.5 (C_ar_), 128.7 (CH_ar_), 131.7 (CH_ar_), 138.2 (C_ar_), 168.6 (C=O) ppm; ^77^Se (76.3 MHz, CDCl_3_), δ = 866.07 ppm; Elemental Anal. Calcd for C_17_H_21_NOSe (334.315): C, 61.08; H, 6.33. Found: C, 61.29; H, 6.40.

*N-Bornyl-1,2-benzisoselenazol-3(2H)-one* (**20**). Yield: 90%, m.p. 228–230 °C, ${\left[\alpha \right]}_{D}^{25}$ = −64.3 (*c* 0.7, CHCl_3_), ^1^H-NMR (700 MHz, CDCl_3_) δ = 0.91 (s, 3H, CH_3_), 0.96 (s, 3H, CH_3_), 1.06 (s, 3H, CH_3_), 1.38–1.44 (m, 3H), 1.78 (t, *J* = 4.2 Hz, 1H), 1.88–1.93 (m, 1H), 1.97–2.01 (m, 1H), 2.45–2.50 (m, 1H), 5.03 (ddd, *J* = 2.1, 4.9, 11.9 Hz, 1H), 7.41 (dt, *J* = 1.4, 7,7 Hz, 1H_ar_), 7.57 (dt, *J* = 1.4, 7,0 Hz, 1H_ar_), 7.60–7.63 (m, 1H), 8.02 (d, *J* = 7.7 Hz, 1H_ar_) ppm; ^13^C-NMR (100.6 Hz, CDCl_3_) δ = 14.2 (CH_3_), 18.9 (CH_3_), 19.9 (CH_3_), 28.3 (CH_2_), 28.6 (CH_2_), 38.1 (CH_2_), 45.2 (CH), 48.4 (C), 52.1 (C), 60.1 (CH), 123.2 (CH_ar_), 126.0 (CH_ar_), 128.3 (C_ar_), 128.7 (CH_ar_), 131.7 (CH_ar_), 138.0 (C_ar_), 168.3 (C=O) ppm; ^77^Se (76.3 MHz, CDCl_3_), δ = 877.38 ppm; Elemental Anal. Calcd for C_17_H_21_NOSe (334.315): C, 61.08; H, 6.33. Found: C, 61.24; H, 6.31.

#### 3.2.7. Conversion of Diselenides to Benzisoselenazolones

To a solution of diselenide **17** and **18** (1.0 mmol) in DMF (3 mL), under argon atmosphere, potassium iodate (1.0 mmol) was added and the mixture was stirred at 120 °C for 24 h. The mixture was cooled to ambient temperature and brine (5 mL) was added. Mixture was stirred for 3 h and the formed precipitate was filtered and dried in air. Yield: 83% (**19**), 86% (**20**).

#### 3.2.8. Synthesis of 2,2-Diselenobis(benzoic acid) **6** and 2-(Chloroseleno)benzoyl Chloride **7**

To a suspension of selenium (12.66 mmol) in water (7 mL) sodium borohydride (25.32 mmol) was added under argon atmosphere. Mixture was stirred for 0.5 h at 0 °C, selenium (12.66 mmol) was added, and stirring was continued at the same temperature for 1 h. Mixture was warmed to room temperature and stirred for 18 h. After adding 10% NaOH (5 mL) the reaction was cooled to 5 °C and the diazonium salt of anthranilic acid was added dropwise. (The diazonium salt was prepared in advance—to a solution of anthranilic acid (25.96 mmol) and concentrated hydrochloric acid (5 mL) in water (15 mL) cooled to 5 °C a cooled solution of sodium nitrite (27.54 mmol) in water (15 mL) was added dropwise and the reaction was stirred for 15 min at 5 °C). The mixture was stirred for 3 h at 60 °C and for 18 h at room temperature. The formed precipitate was filtred off and the solution was acidified to pH = 1 by 36% HCl. The formed precipitate was filtrated. The crude product **6** was purified by washing with boiling water and dried in air. Acid 6 (0.01 mmol) was further converted to 2-(chloroseleno)benzoyl chloride **7** by heating with thionyl chloride (20 mL) at 85 °C for 3 h. Thionyl chloride was distilled off, and the crude product was used without further purification.

*2,2-Diselenobis(benzoic acid)* (**6**). Yield: 90%, ^1^H-NMR (700.27 MHz, DMSO) δ = 7.35–7.37 (m, 2H_ar_), 7.48–7.50 (m, 2H_ar_), 7.67 (dd, *J* = 8.4, 0.7, 2H, 2× CH_ar_), 8.03 (dd, *J* = 7.7, 1.4, 2H, 2× CH_ar_), 13.72 (s, 2H, 2× OH); ^13^C-NMR (100.6 Hz, DMSO) δ = 126.9 (2× CH_ar_), 129.4 (2× C_ar_), 129.9 (2× CH_ar_), 131.9 (2× CH_ar_), 133.93 (2× C_ar_), 133.97 (2× CH_ar_), 169.0 (2× C=O) ppm; ^77^Se (76.3 MHz, DMSO), δ = 441.09 ppm.

*2-(Chloroseleno)benzoyl chloride* (**7**). Yield 98%; ^1^H-NMR (400.13 MHz, CDCl_3_) δ = 7.50–7.54 (m, 1H_ar_), 7.80–7.84 (m, 1H_ar_), 8.12 (dd, *J* = 8.4, 0.8, 1H, CH_ar_), 8.37 (dd, *J* = 8.0, 1.2, 1H, CH_ar_); ^13^C-NMR (CDCl_3_, 100.62 MHz) δ = 126.7 (CH_ar_), 127.1 (CH_ar_), 129.0 (C_ar_), 134.5 (CH_ar_), 136.2 (CH_ar_), 146.3 (C_ar_), 172.6 (C_ar_); ^77^Se-NMR (CDCl_3_, 76.35 MHz) δ = 1060.46 ppm.

#### 3.2.9. General Procedure for the Synthesis of Compounds **21**–**25**

To a solution of amine (2.0 mmol) and triethylamine (4.0 mmol) in dichloromethane 2-(chloroseleno)benzoyl chloride (1.0 mmol) was added. The mixture was stirred for 24 h at room temperature, poured on water and extracted with DCM. The combined organic layers were dried over anhydrous magnesium sulfate and evaporated [22].

*N-(Glycine methyl ester)-benzisoselenazol-3(2H)-one* (**21**). Yield 68%; ^1^H-NMR (400.13 MHz, CDCl_3_) δ = 3.80–3.81 (m, 3H), 4.61–4.62 (d, *J* = 4.0, 2H), 7.43–7.45 (m, 1H_ar_), 7.61–7.68 (m, 2H_ar_), 8.06–8.08 (m, 1H_ar_); ^13^C-NMR (176.10 MHz, CDCl_3_) δ = 45.0 (CH_2_), 52.2 (-OCH_3_), 123.6 (CH_ar_), 125.6 (C_ar_), 125.9 (CH_ar_), 128.6 (CH_ar_), 132.0 (CH_ar_), 138.5 (C_ar_), 167.4 (C=O), 168.7 (C=O); ^77^Se-NMR (76.35 MHz, CDCl_3_) δ = 937.49 ppm.

*N-(Alanine methyl ester)-benzisoselenazol-3(2H)-one* (**22**). Yield; 84%; ^1^H-NMR (700.27 MHz, CDCl_3_) δ = 1.58 (d, *J* = 7.0, 3H, CH_3_), 3.74 (s, 3H, -OCH_3_), 5.42 (q, *J* = 7.0, 1H, CH), 7.40–7.42 (m, 1H_ar_), 7.58–7.60 (m, 1H_ar_), 7.64–7.65 (m, 1H_ar_), 8.05 (d, *J* = 7.7, 1H, CH_ar_); ^13^C-NMR (100.62 MHz, CDCl_3_) δ = 18.7 (CH_3_), 51.6 (-OCH_3_), 52.7 (CH), 123.8 (CH_ar_), 126.2 (CH_ar_), 126.9 (C_ar_), 128.8 (CH_ar_), 132.2 (CH_ar_), 139.2 (C_ar_), 167.5 (C=O), 172.2 (C=O); ^77^Se-NMR (76.35 MHz, CDCl_3_) δ = 888.79 ppm.

*N-(Leucine methyl ester)-benzisoselenazol-3(2H)-one* (**23**). Yield 83%; ^1^H-NMR (700.27 MHz, CDCl_3_) δ = 0.96 (d, *J* = 6.3, 3H, CH_3_) 0.97 (d, *J* = 7, 3H, CH_3_), 1.53–1.59 (m, 1H), 1.75–1.85 (m, 2H), 3.75 (s, 3H, -OCH_3_), 5.44–5.46 (m, 1H), 7.40–7.42 (m, 1H_ar_), 7.59 (dt, *J* = 7.0, 1.4, 1H, CH_ar_), 7.63 (d, *J* = 7.7, 1H, CH_ar_), 8.05 (dd, *J* = 7.7, 0.7, 1H, CH_ar_); ^13^C-NMR (176.10 MHz, CDCl_3_) δ = 22.6 (CH_3_), 23.9 (CH_3_), 25.6 (CH), 43.2 (CH_2_), 53.5 (-OCH_3_), 55.2 (CH), 124.9 (CH_ar_), 127.1 (CH_ar_), 127.9 (C_ar_), 129.9 (CH_ar_), 133.2 (CH_ar_), 140.0 (C_ar_), 168.6 (C=O), 173.1 (C=O); ^77^Se-NMR (76.35 MHz, CDCl_3_) δ = 886.93 ppm.

*N-(Phenylalanine methyl ester)-benzisoselenazol-3(2H)-one* (**24**). Yield 98%; ^1^H-NMR (700.27 MHz, CDCl_3_) δ = 3.15–3.18 (m, 1H), 3.33–3.36 (m, 1H), 3.70 (s, 3H, -OCH_3_), 5.62–5.64 (m, 1H), 7.19–7.261 (m, 5H), 7.38 (dt, *J* = 7.0, 0.7, 1H, CH_ar_), 7.56–7.62 (m, 2H_ar_), 8.00 (dd, *J* = 7.7, 0.7, 1H, CH_ar_); ^13^C-NMR (100.62 MHz, CDCl_3_) δ = 39.1 (CH_2_), 52.5 (-OCH_3_), 57.2 (CH), 123.7 (CH_ar_), 126.1 (CH_ar_), 126.6 (C_ar_), 127.2 (CH_ar_), 128.6 (2× CH_ar_), 128.8 (CH_ar_), 129.1 (2× CH_ar_), 132.2 (CH_ar_), 135.5 (C_ar_), 139.3 (C_ar_), 167.5 (C=O), 171.4 (C=O); ^77^Se-NMR (76.35 MHz, CDCl_3_) δ = 904.16 ppm.

*N-(Alanine carboxyl acid)-benzisoselenazol-3(2H)-one* (**25**). Yield 63%; ^1^H-NMR (400.13 MHz, CDCl_3_) δ = 2.73–2.78 (m, 2H), 4.10 (t, *J* = 6.4, 2H, CH_2_), 7.44 (t, *J* = 7.2, 1H, CH_ar_), 7.60–7.64 (m, 1H_ar_), 7.91–7.94 (m, 2H_ar_); ^13^C-NMR (100.62 MHz, CDCl_3_) δ = 34.0 (CH_2_), 40.1 (CH_2_), 124.8 (CH_ar_), 125.7 (CH_ar_), 127.2 (C_ar_), 127.3 (CH_ar_), 131.7 (CH_ar_), 168.0 (C=O), 172.1 (C=O); ^77^Se-NMR (76.35 MHz, CDCl_3_) δ = 922.33 ppm.

### 3.3. Antioxidant Activity Assay

To a solution of compounds and **17**–**25** (0.015 mmol) and dithiothreitole DTT^red^ (0.15 mmol) in 1.1 mL of CD_3_OD 30% H_2_O_2_ (0.15 mmol) was added. ^1^H-NMR spectra were measuared right after addition of hydrogen peroxide and then in specific time intervals. The concentration of the substrate was determined according to the changes in the integration on the ^1^H-NMR spectra [43].

### 3.4. Cell Viability Assays

#### 3.4.1. MTT Viability Assay

##### Cell Culture

The HL-60, MCF-7, and HepG2 cell lines were purchased from the European Collection of Cell Cultures (ECACC, Salisbury, UK). HL-60 cells were cultured in RPMI 1640 plus GlutaMax I medium (Invitrogen, Grand Island, NY, USA), supplemented with 10% heat-inactivated FBS (Biological Industries, Beit-Haemek, Israel) and antibiotics (100 μg/mL streptomycin and 100 U/mL penicillin) (Sigma Aldrich, St. Louis, MO, USA). MCF-7 and HepG2 cells were cultured in MEME (Sigma–Aldrich, St. Louis, MO, USA), supplemented with 10% FBS (Biological Industries, Beit-Haemek, Israel), 2 mM glutamine, Men Non-essential amino acid solution and antibiotics (100 mg/mL streptomycin and 100 U/mL penicillin), all from Sigma Aldrich (St. Louis, MO, USA). Cells were maintained at 37 °C in a 5% CO_2_ atmosphere and were grown until 80% confluent.

##### MTT Assay

The MTT (3-(4,5-dimethyldiazol-2-yl)-2,5 diphenyl tetrazolium bromide) assay, which measures activity of cellular dehydrogenases, was based on the method of Mosmann [46]. Briefly, cells were seeded into 96-well plates (about 1.5 × 104 cells per well, in 100 µL) and then left to adhere and grow for 24 h. Subsequently, 100 µL of the tested compounds in the medium were added to a final concentration of 0–250 μM, and kept for 48 h, followed by the addition of 100 µL MTT, 3 mg/mL in PBS, for the next 3 h. After the incubation, the medium was removed. Remaining insoluble formazan crystals were dissolved in 100 µL DMSO. The absorbance of the blue formazan product was measured at 570 nm in the plate reader spectrophotometer Infinite M200 (Tecan, Grödig, Austria) and compared with control (untreated cells). All experiments were performed three times in triplicate. The concentration of tested compounds required to inhibit cell viability by 50% (IC_50_) was calculated using Microsoft Excel software for semi-log curve fitting with linear regression analysis.

#### 3.4.2. SRB Viability Assay

##### Cell Culture

The prostate cancer cell line DU-145 was purchased from the American Type Culture Collection (ATTC, Manassas, VA, USA). The DU-145 cells were cultured in MEME medium supplemented with 10% fetal bovine serum, 1% penicillin/streptomycin, 2 mM glutamine and 1 mM sodium pyruvate at 37 °C. The PNT1A cells were cultured in RPMI 1640 supplemented with serum, l-glutamine and antibiotics. The cells were maintained at 37 °C in an atmosphere containing 5% CO_2_. Stock solutions of *N*-substituted ebselen derivatives were prepared in (0.1%) DMSO.

##### SRB Assay

Cell viability was measured by Sulphorhodamine B (SRB) assay. The cells were grown to sub-confluent levels at the certain culture medium and then seeded into 96-well plates at 6 × 10^3^ cells/well in the final volume of 200 µL in the culture medium for 24 h. Then, they were treated with various concentrations (2.5, 5, 10, 20, 30, and 40 μM) of *N*-substituted ebelsen derivatives for the next 24 h. After incubation, the cells were fixed in 20% trichloroacetic acid for an 1 h. The plates were washed with distilled water, and 0.4% SRB (Sigma-Aldrich, St. Louis, MO, USA) in 1% acetic acid solution was added to the plates for 15 min. The SRB solution was washed with 1% acetic acid. SRB was then solubilized in 10 mM Trisma-base solution and the absorbance was measured at 570 nm using an automated microplate reader. The experiments were done in triplates and the IC_50_ values were calculated.

## 4. Conclusions

In this paper, we synthetized the first optically active ebselen analogues derived from terpenes **17**–**20**. Additionally, we presented an improved version of the known procedure involving the synthesis of 2,2-diselenobis(benzoic acid) **6** and proved that the cyclization of the Se-N bond depends on the nucleophilic selenium reagent used. Antioxidant and anticancer activities of *N*-bornyl **20** and *N*-isobornyl **19** benzisoselenazol-3(2*H*)-ones and corresponding diselenides **17**, **18** were evaluated and compared to a series of *N*-amino acid ebselen analogues **21**–**25**. The highest antioxidant potential was observed for benzisoselenazol-3(2*H*)-ones substituted with *N*-bornyl **20** and *N*-leucine methyl ester **23** moieties. Select reactive derivatives were tested as anticancer agents on four cancer cell lines. The highest antiproliferative activity was observed for *N*-leucine methyl ester benzisoselenazol-3(2*H*)-one **23**. Additionally, we tested antiproliferative activity for ebselen with 3-methylbutyl carbon chain **26**. The data suggest that the basic structural skeleton connecting 3-methylbutyl carbon chain can be a pharmacophore necessary for the antioxidant and anticancer activity.

## Figures and Tables

**Table 1:**
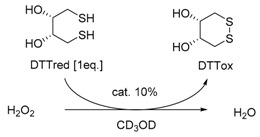
Results of the antioxidant activity measurement.

	Remaining Dithiotreitol (%)				
Catalyst	3 min	5 min	15 min	30 min	60 min
**17**	59	36	12	0	0
**18**	48	38	28	25	22
**19**	50	17	0	0	0
**20**	30	11	0	0	0
**21**	99	99	98	97	95
**22**	96	95	93	91	89
**23**	45	0	0	0	0
**24**	96	95	94	93	90
**25**	94	93	92	92	91
**26**	77	58	42	28	13
**Ebselen (1)**	84	75	64	58	52
**Diselenide (3)**	89	83	74	68	63

**Table 2:** Results of the cytotoxic activity assays.

Comp.	Structure	MCF-7 (MTT)	HEP G2 (MTT)	HL60 (MTT)	DU-145 (SRB)	PNT1A (SRB)
		IC_50_, µM				
**20**	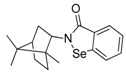	220 ± 34	232 ± 28	190 ± 20	>40	>40
**23**	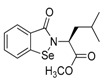	18 ± 1.1	23.5 ± 3.2	21.5 ± 1.8	>40	>40
**26**	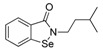	29 ± 1.9	100 ± 12	43.5 ± 5.0	30.6 ± 0.2	30.35 ± 0.06
**Control**	Carboplatin	0.70 ± 0.30	3.19 ± 0.46	9.70 ± 1.20	-----	------

## Supplementary Materials

Supplementary materials are available online.
