# Supplementary material for: New Chiral Ebselen Analogues with Antioxidant and Cytotoxic Potential
Source: Molecules. 2017 Mar 20;22(3):492. doi: 10.3390/molecules22030492 (PMC6155185; doi:10.3390/molecules22030492)
Supplement: Supplementary file 1 [file molecules-22-00492-s001.pdf]

New chiral ebselen analogues with antioxidant and cytotoxic potential

A. J. Pacuła[a], K. B. Kaczor[b], J. Antosiewicz[b,e], A. Janecka[c], A. Długosz[c], T. Janecki[d] , A. Wojtczak[a] and J. Ścianowski[a*]

[a]Department of Organic Chemistry, Faculty of Chemistry, Nicolaus Copernicus University, Gagarina 7, 87-100 Torun, Poland
[b]Department of Bioenergetics and Physiology of Exercise, Medical University of Gdansk, Debinki 1, 80-211 Gdansk, Poland
[c]Department of Biomolecular Chemistry, Faculty of Medicine, Medical University of Lodz, Mazowiecka 6/8, 92-215 Lodz, Poland
[d]Institute of Organic Chemistry, Lodz University of Technology, Zeromskiego 116, 90-924 Lodz, Poland
[e]Department of Biochemistry, Gdansk University of Physical Education and Sport, Kazimierza Gorskiego 1, 80-336 Gdansk, Poland

**E-mail: jsch@chem.umk.pl*

Supporting Information: NMR Spectra, Crystallografic data

Index of content

I. NMR spectra
   a) (1*R*)- Camphor oxime (**10**)
   b) (1*R*)-Camphor *O*-methyloxime (**11**)
   c) (*R*)-(-)-Isobornylamine (**12**)
   d) *N*-Isobornyl-*o*-iodobenzamide (**15**)
   e) *N*-Bornyl-*o*-iodobenzamide (**16**)
   f) 2,2‘-Diselenobis(*N*-isobornylbenzamide) (**17**)
   g) 2,2‘-Diselenobis(*N*-bornylbenzamide) (**18**)
   h) *N*-Isobornyl-1,2-benzisoselenazol-3(2*H*)-one (**19**)
   i) *N*-Bornyl-1,2-benzisoselenazol-3(2*H*)-one (**20**)
   j) 2,2-Diselenobis(benzoic acid) (**6**)
   k) 2-(Chloroseleno)benzoyl chloride (**7**)
   l) *N*-(Glycine methyl ester)-benzisoselenazol-3(2*H*)-one (**21**)
   m) *N*-(Alanine methyl ester)-benzisoselenazol-3(2*H*)-one (**22**)
   n) *N*-(Leucine methyl ester)-benzisoselenazol-3(2*H*)-one (**23**)
   o) *N*-(Phenylalanine methyl ester)-benzisoselenazol-3(2*H*)-one (**24**)
   p) *N*-(Alanine carboxyl acid)-benzisoselenazol-3(2*H*)-one (**25**)

II. Crystallographic data
   a) Table 1. Crystal data and structure refinement for *N*-bornyl-1,2-benzisoselenazol-3(2*H*)-one **20** (e654a).
   b) Table 2. Selected bond lengths [Å] and angles [º] for *N*-bornyl-1,2-benzisoselenazol-3(2*H*)-one **20** (e654a).

I. **NMR spectra**

(1*R*)- Camphor oxime (10)

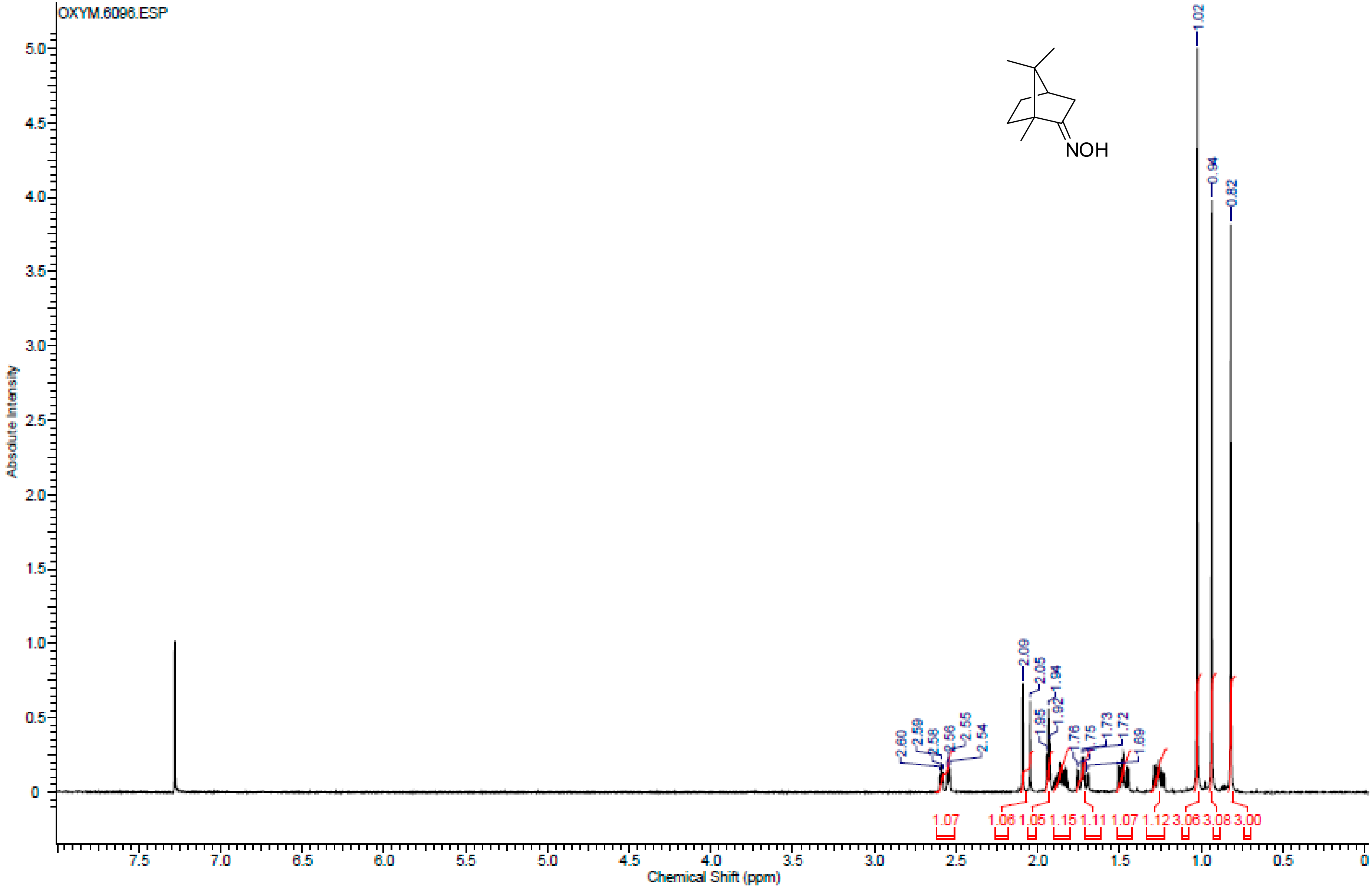

(1*R*)-Camphor *O*-methyloxime (11)

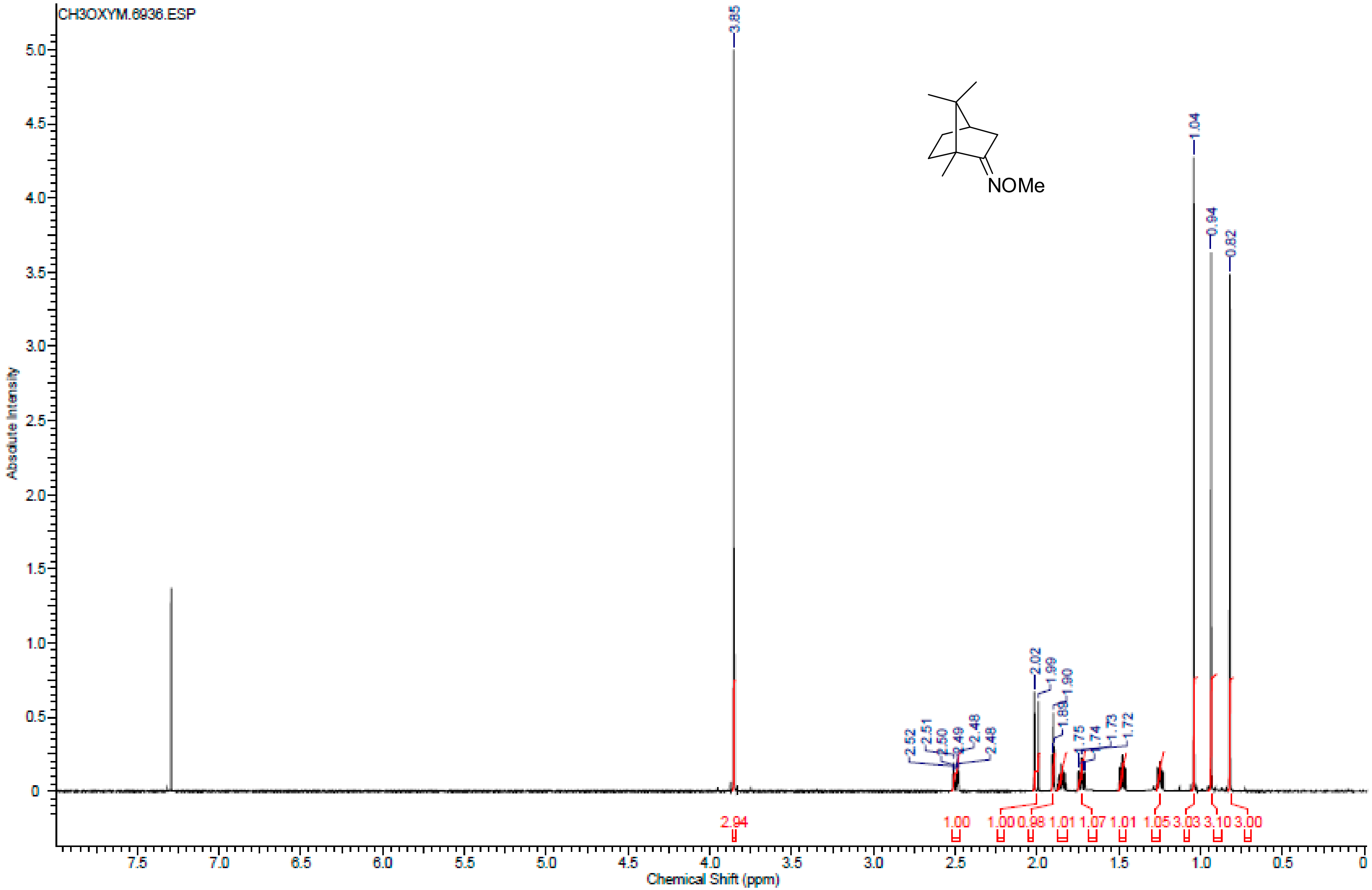

(*R*)-(-)-Isobornylamine (12)

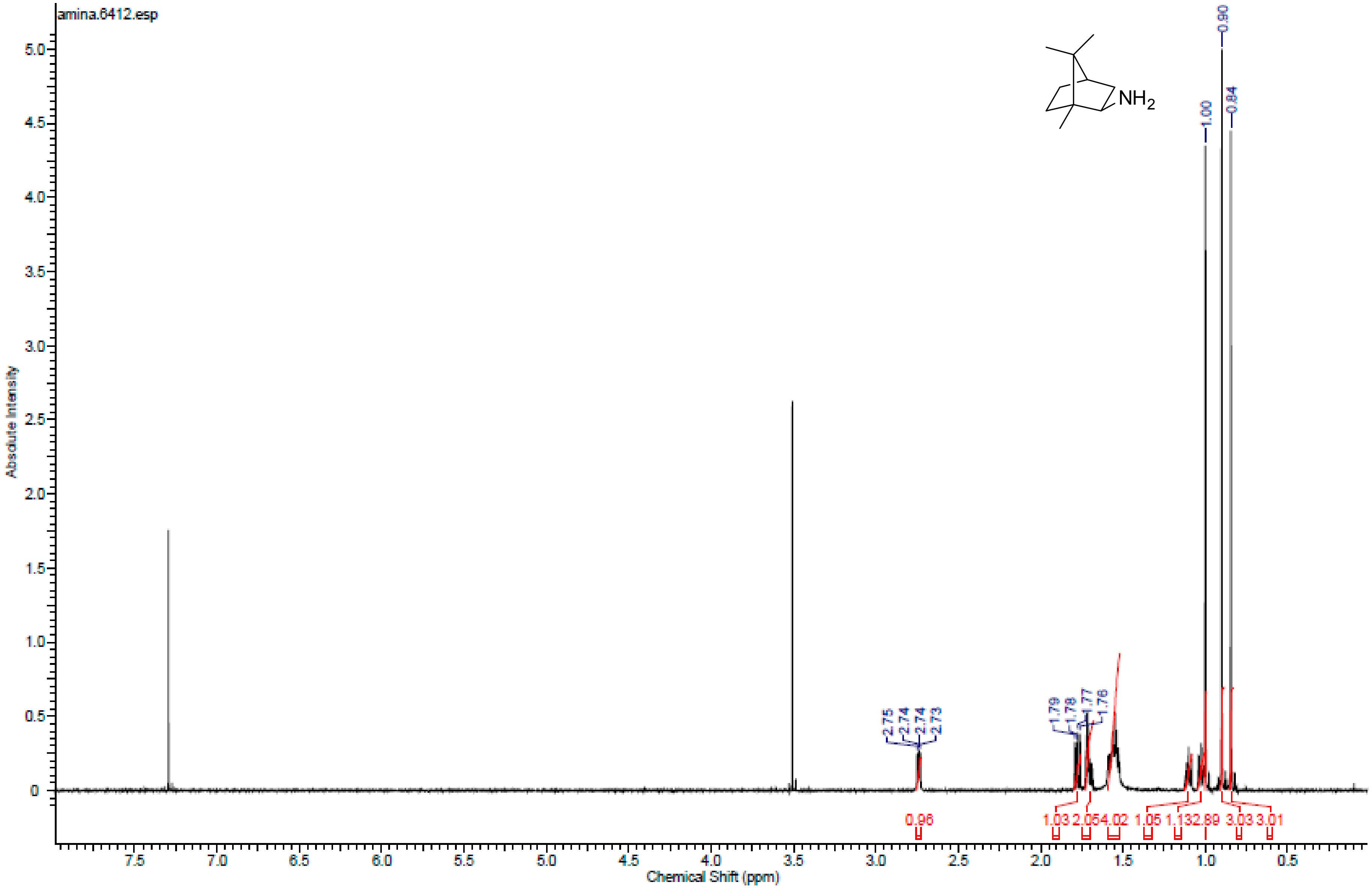

_N_-Isobornyl-_o_-iodobenzamide (15)

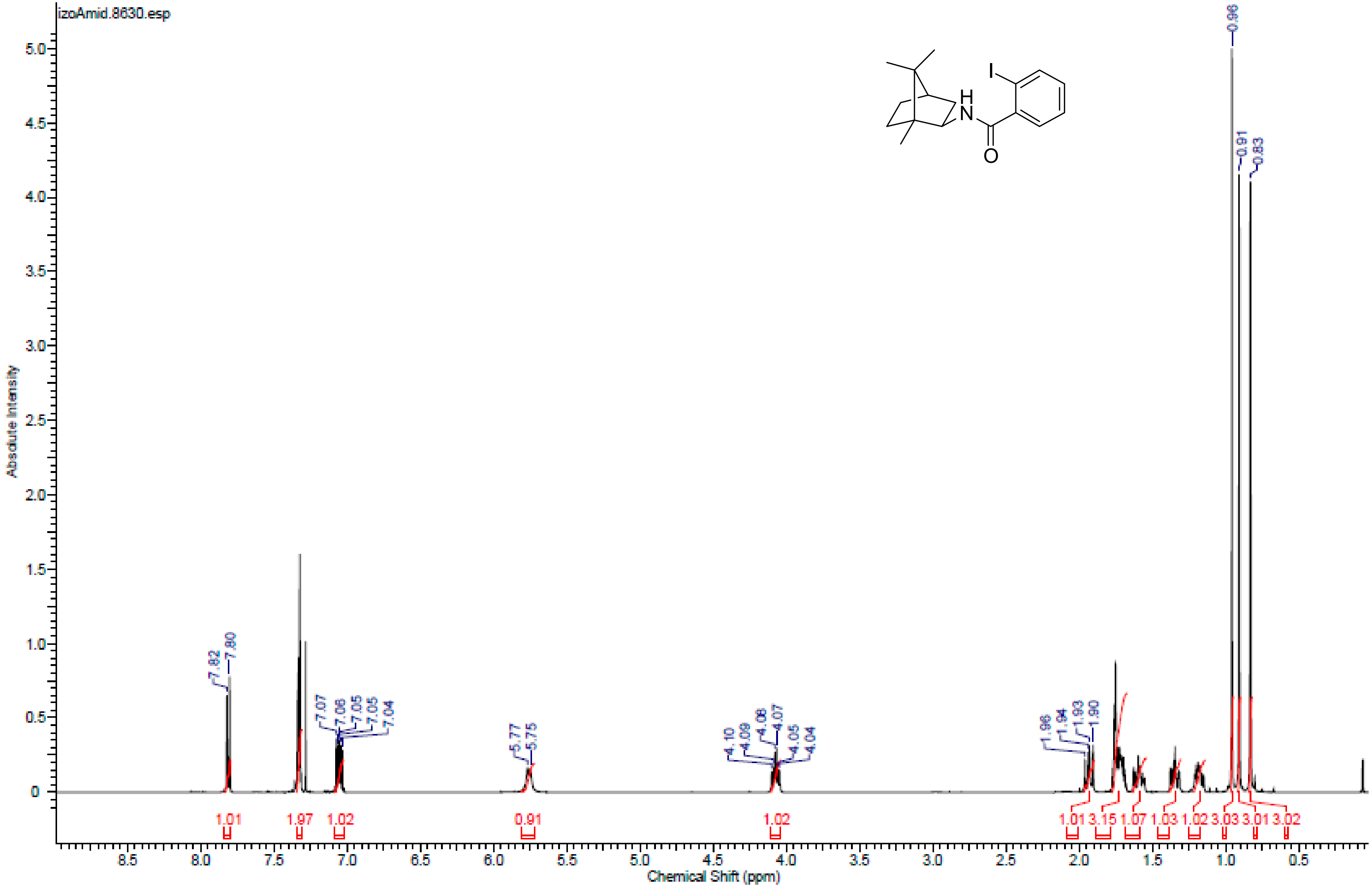

***N*-Isobornyl-*o*-iodobenzamide (15)**

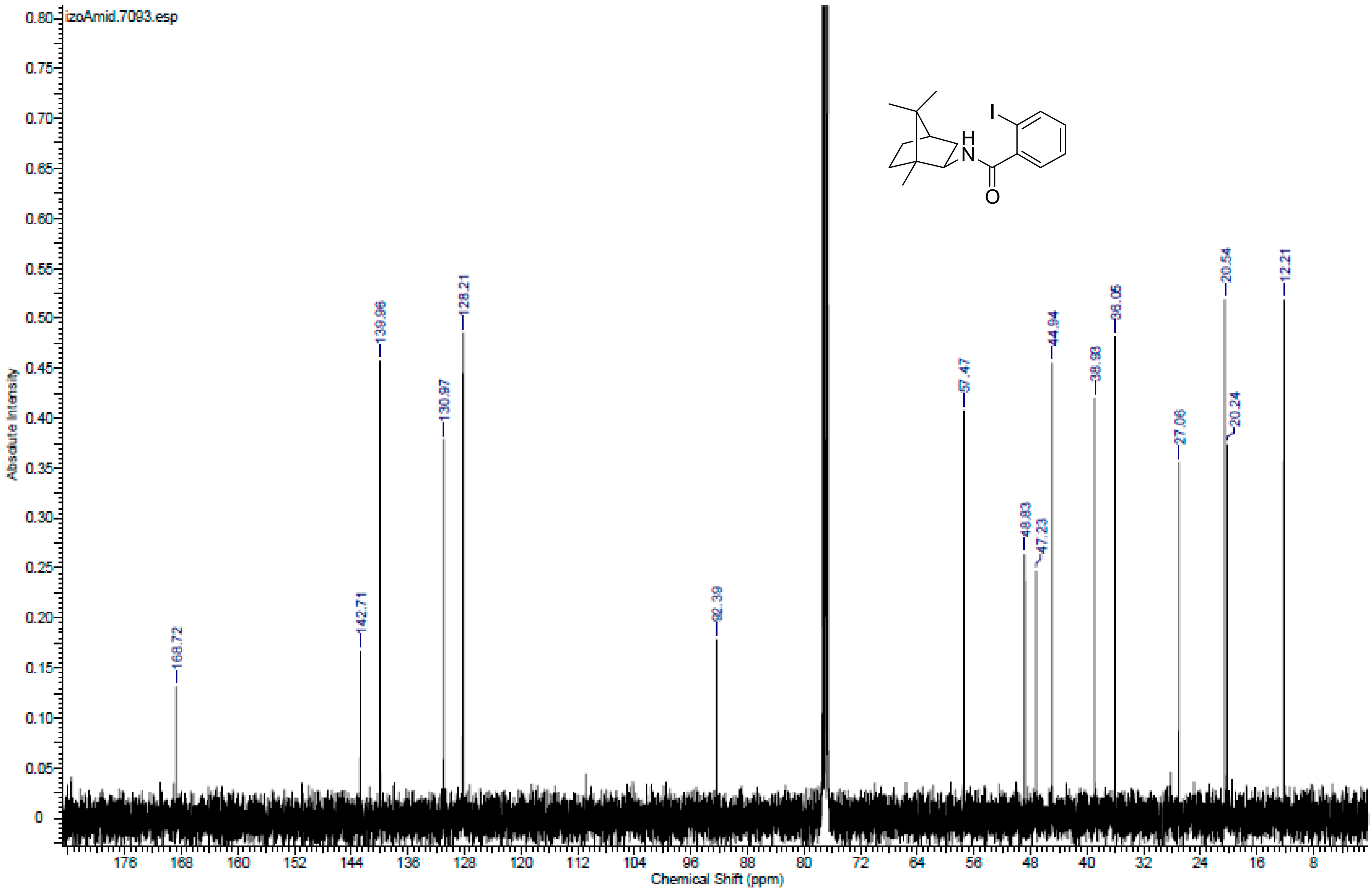

***N*-Bornyl-*o*-iodobenzamide (16)**

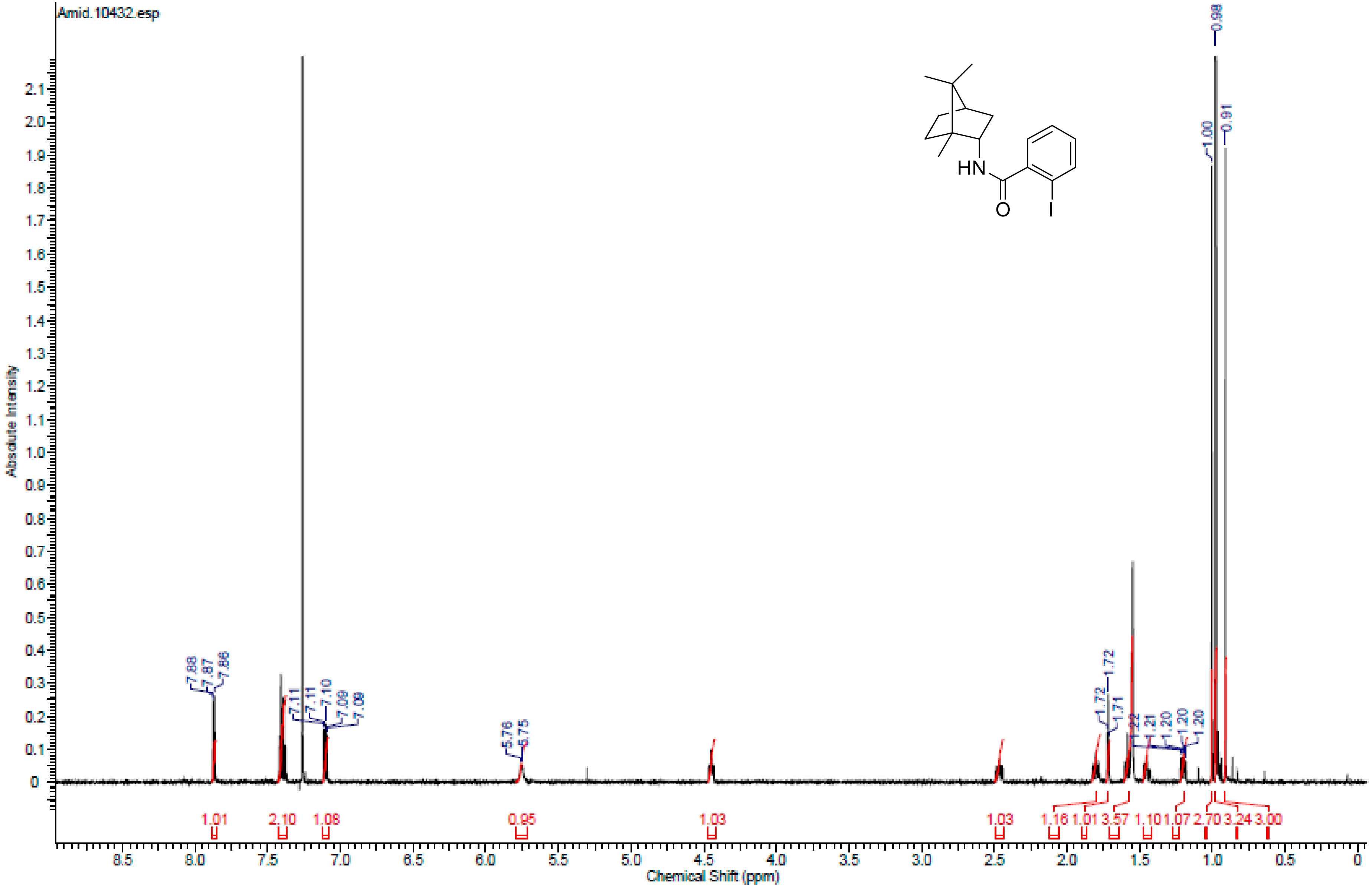

***N*-Bornyl-*o*-iodobenzamide (16)**

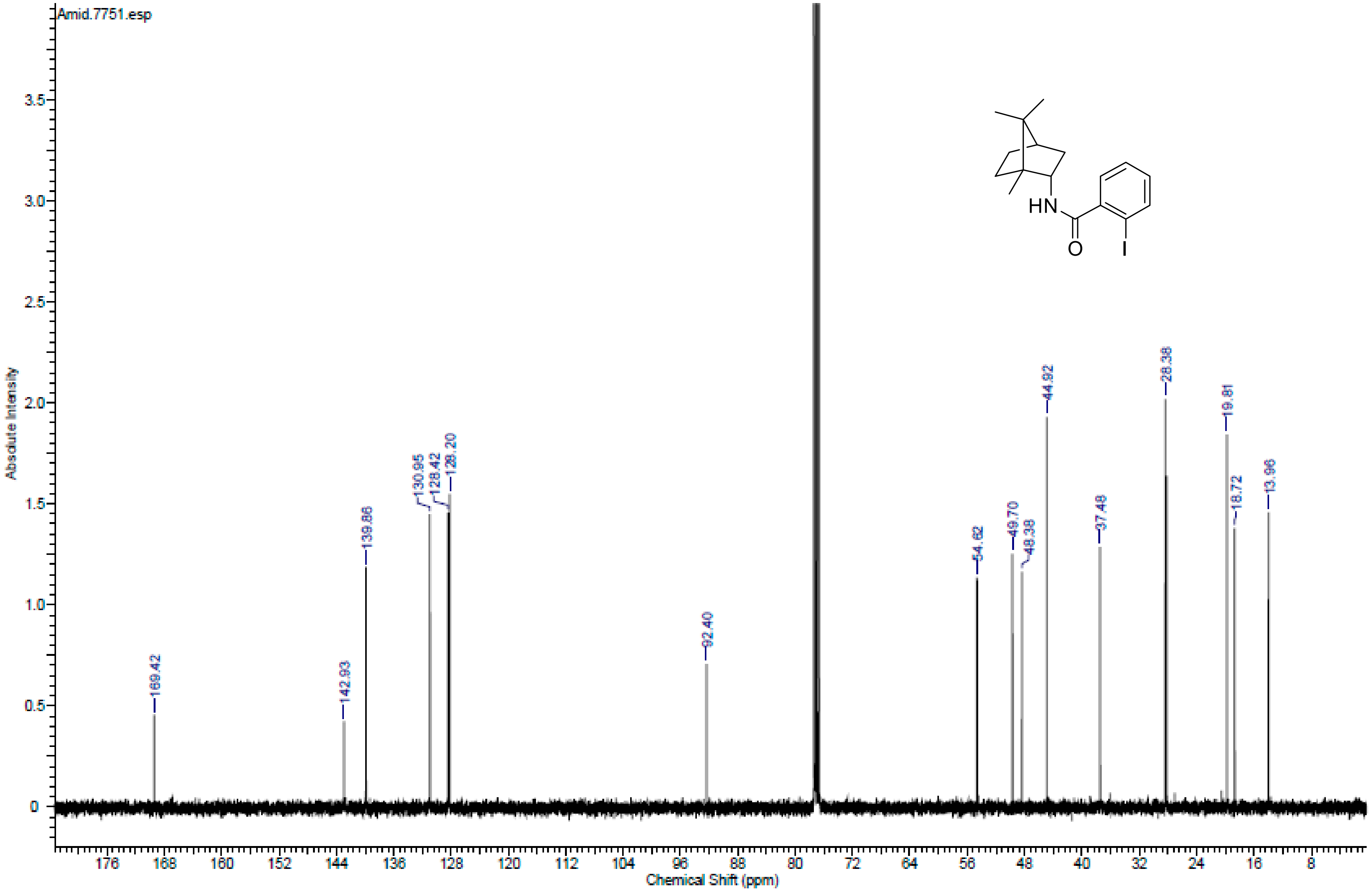

2,2‘-Diselenobis(*N*-isobornylbenzamide) (17)

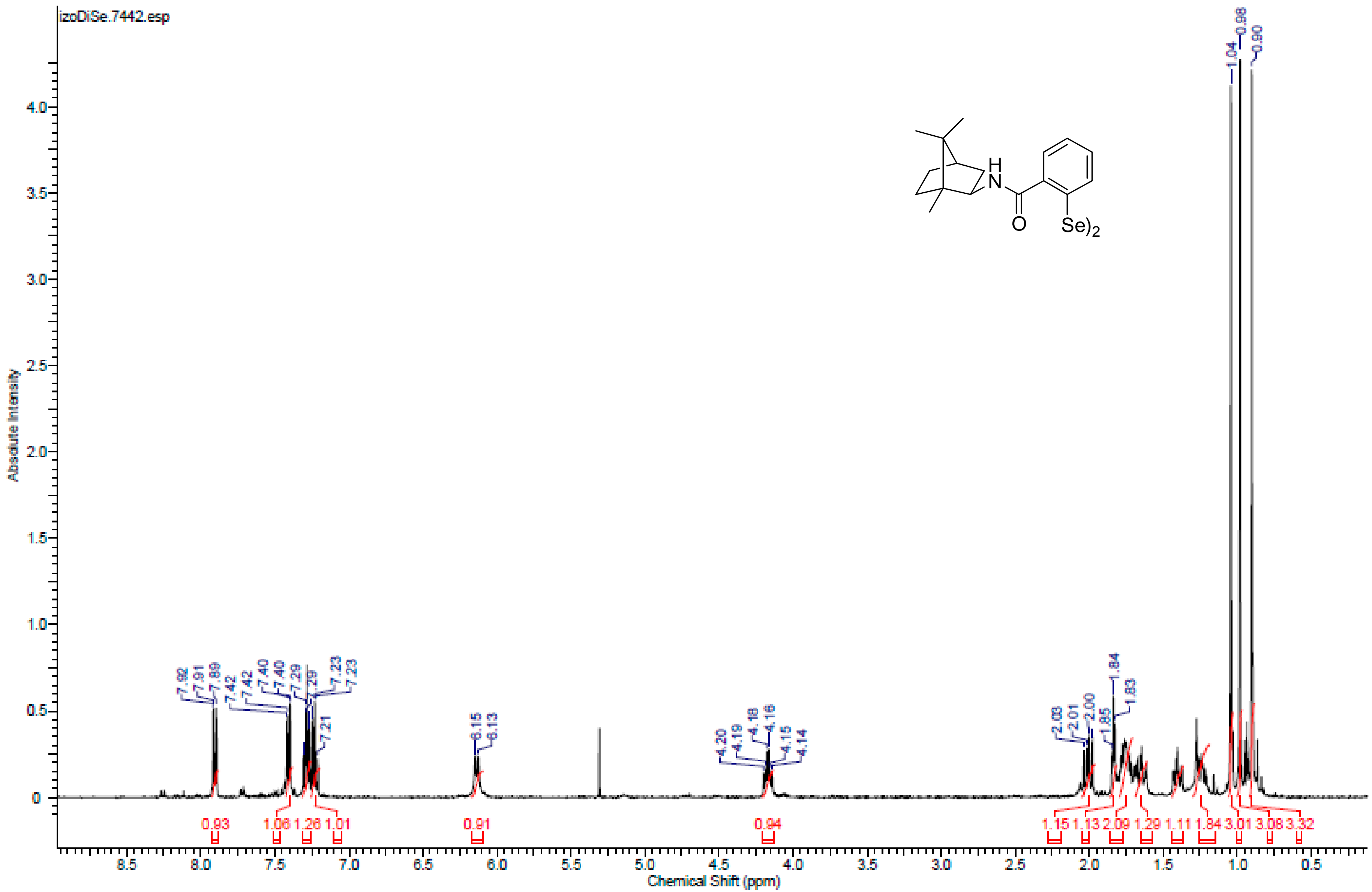

2,2'-Diselenobis(*N*-isobornylbenzamide) (17)

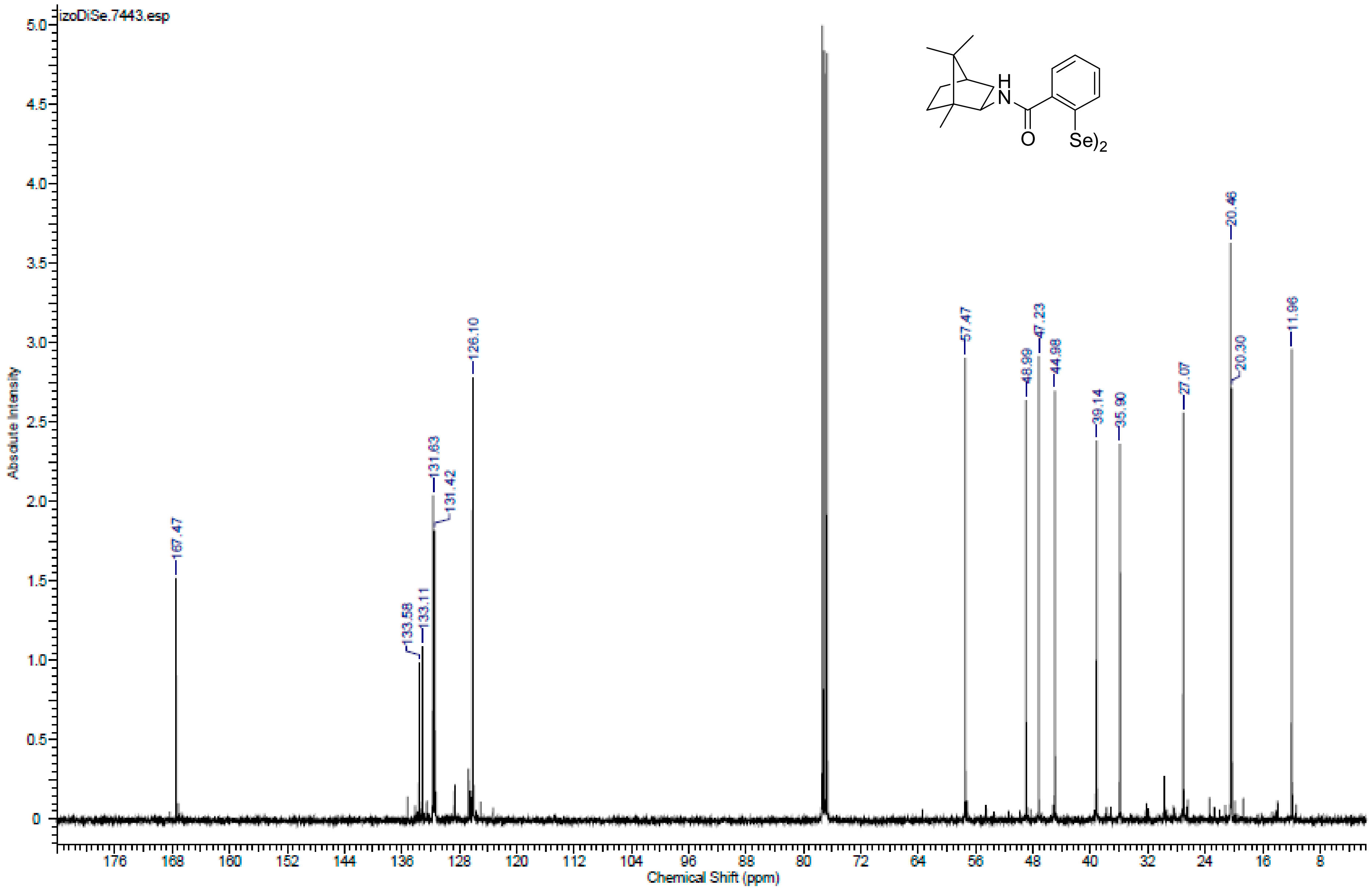

2,2'-Diselenobis(*N*-isobornylbenzamide) (17)

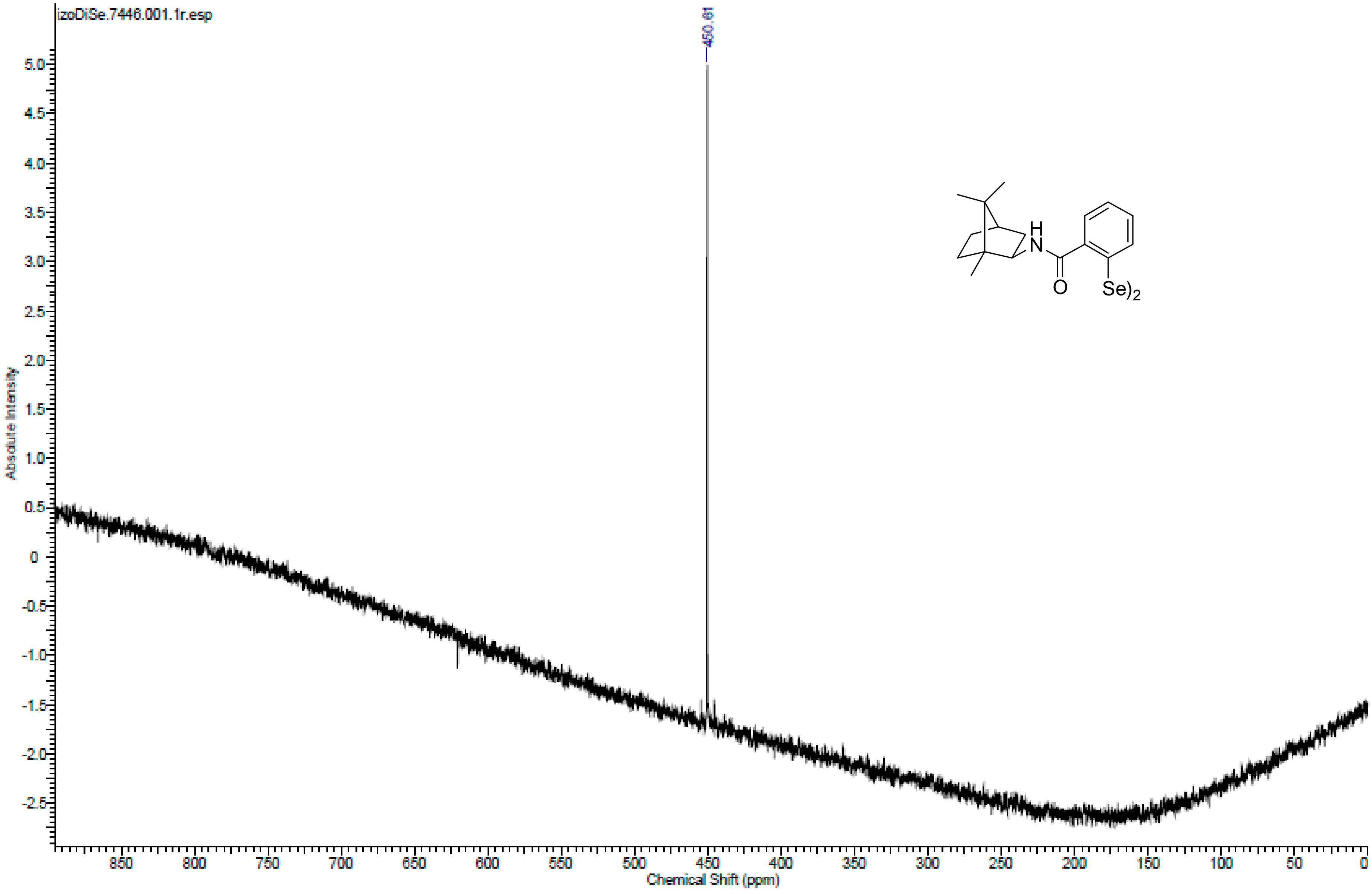

2,2‘-Diselenobis(*N*-bornylbenzamide) (18)

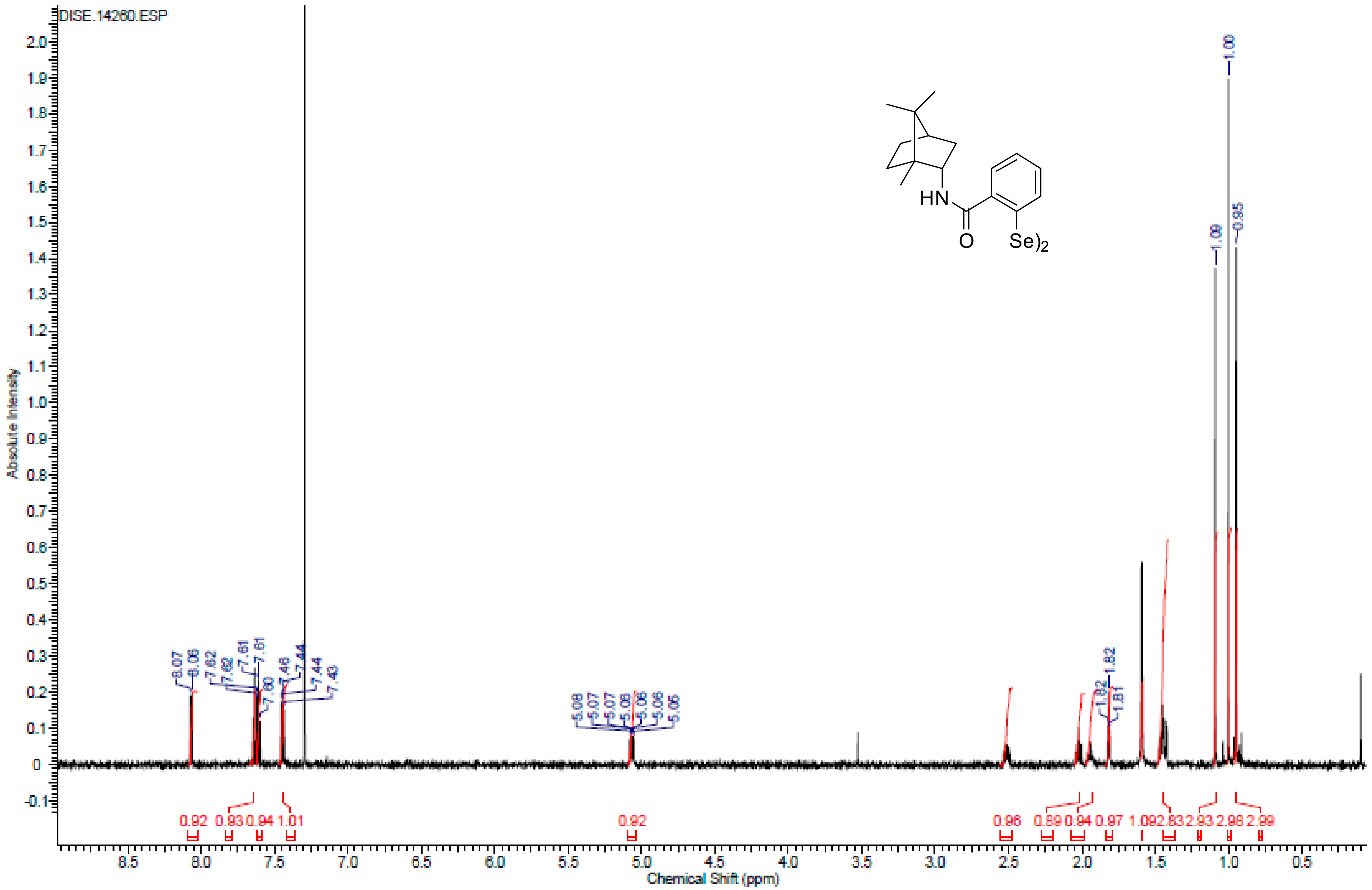

2,2'-Diselenobis(*N*-bornylbenzamide) (18)

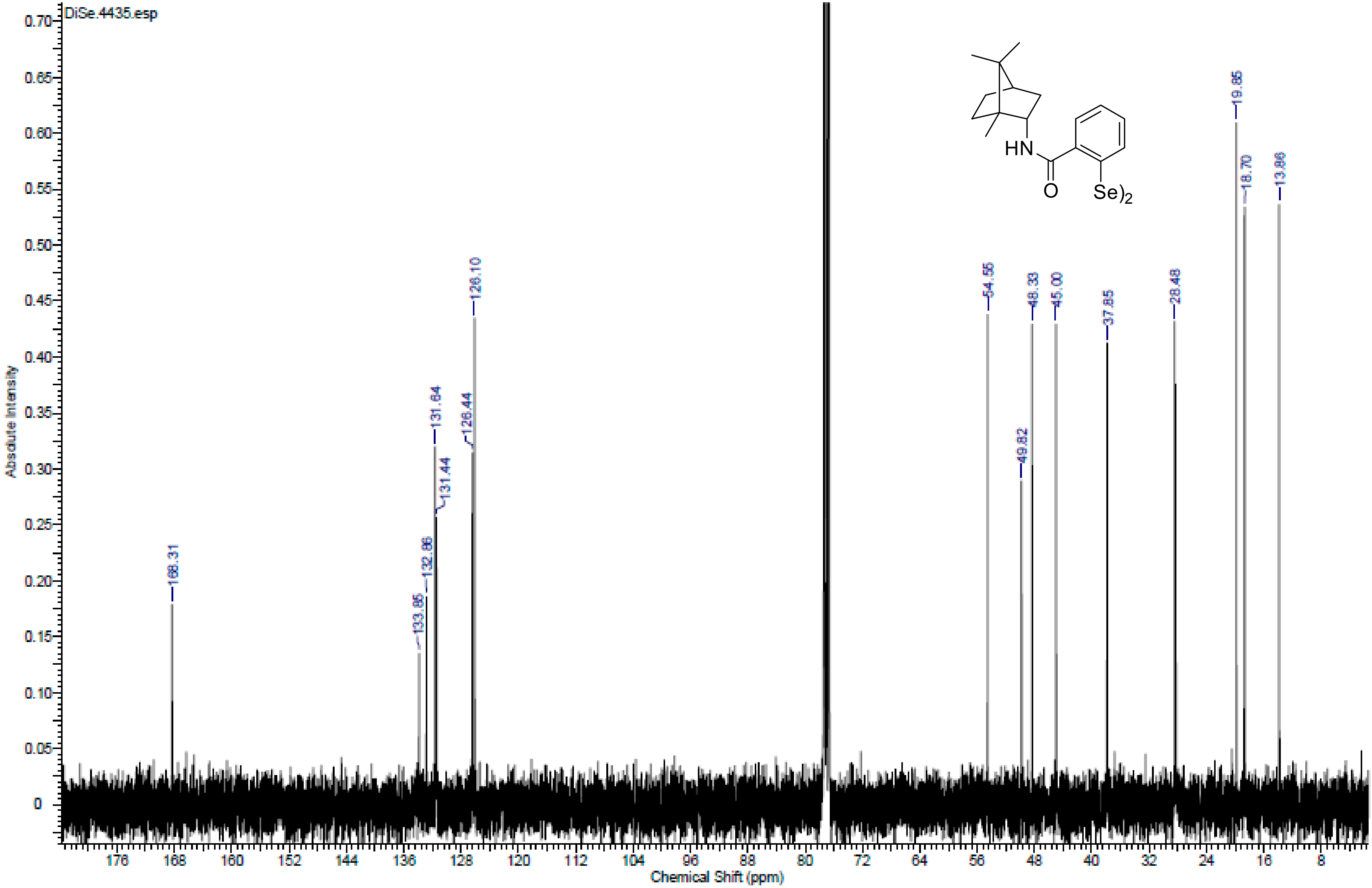

2,2'-Diselenobis(*N*-bornylbenzamide) (18)

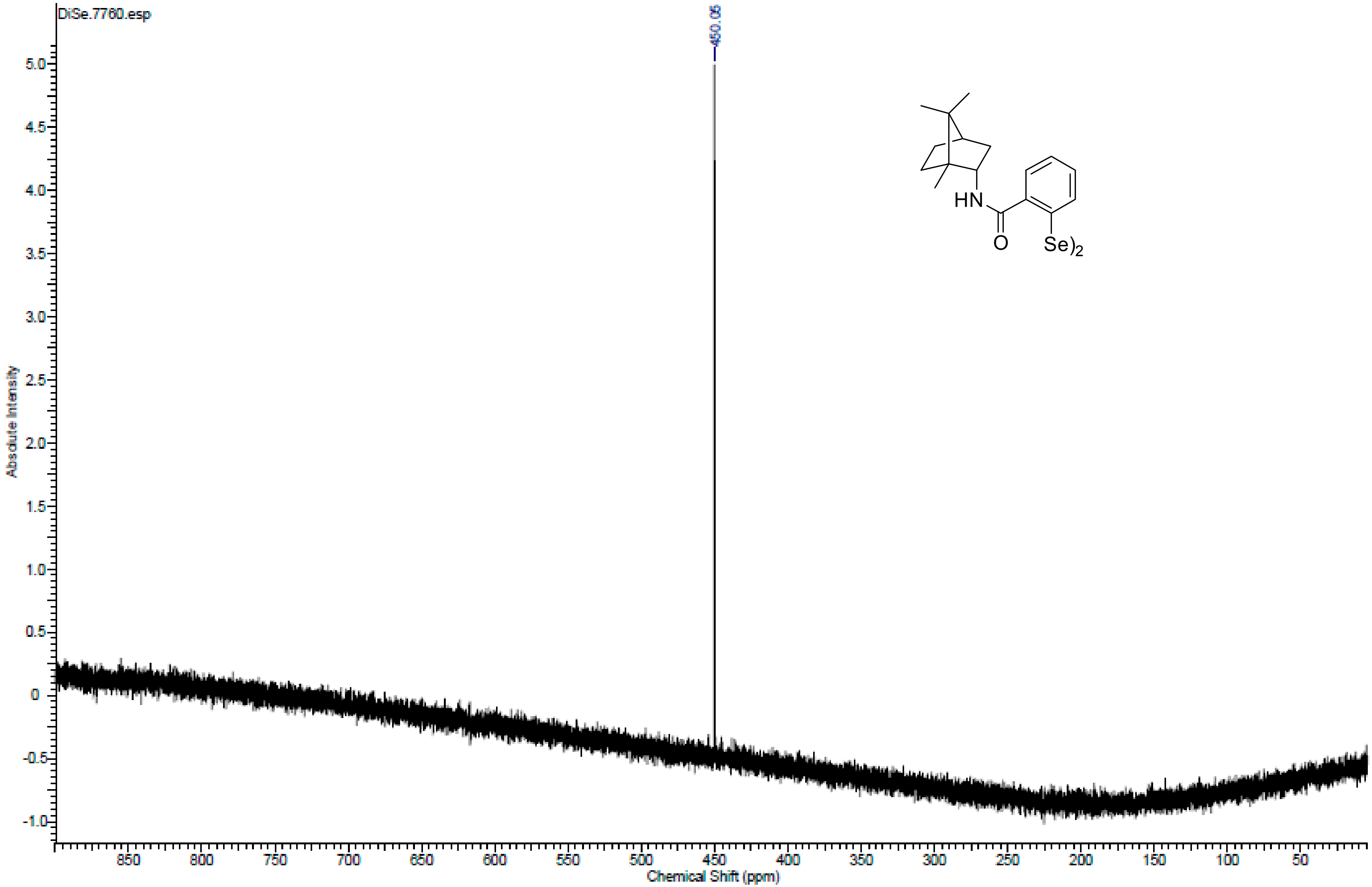

***N*-Isobornyl-1,2-benzisoselenazol-3(2*H*)-one (19)**

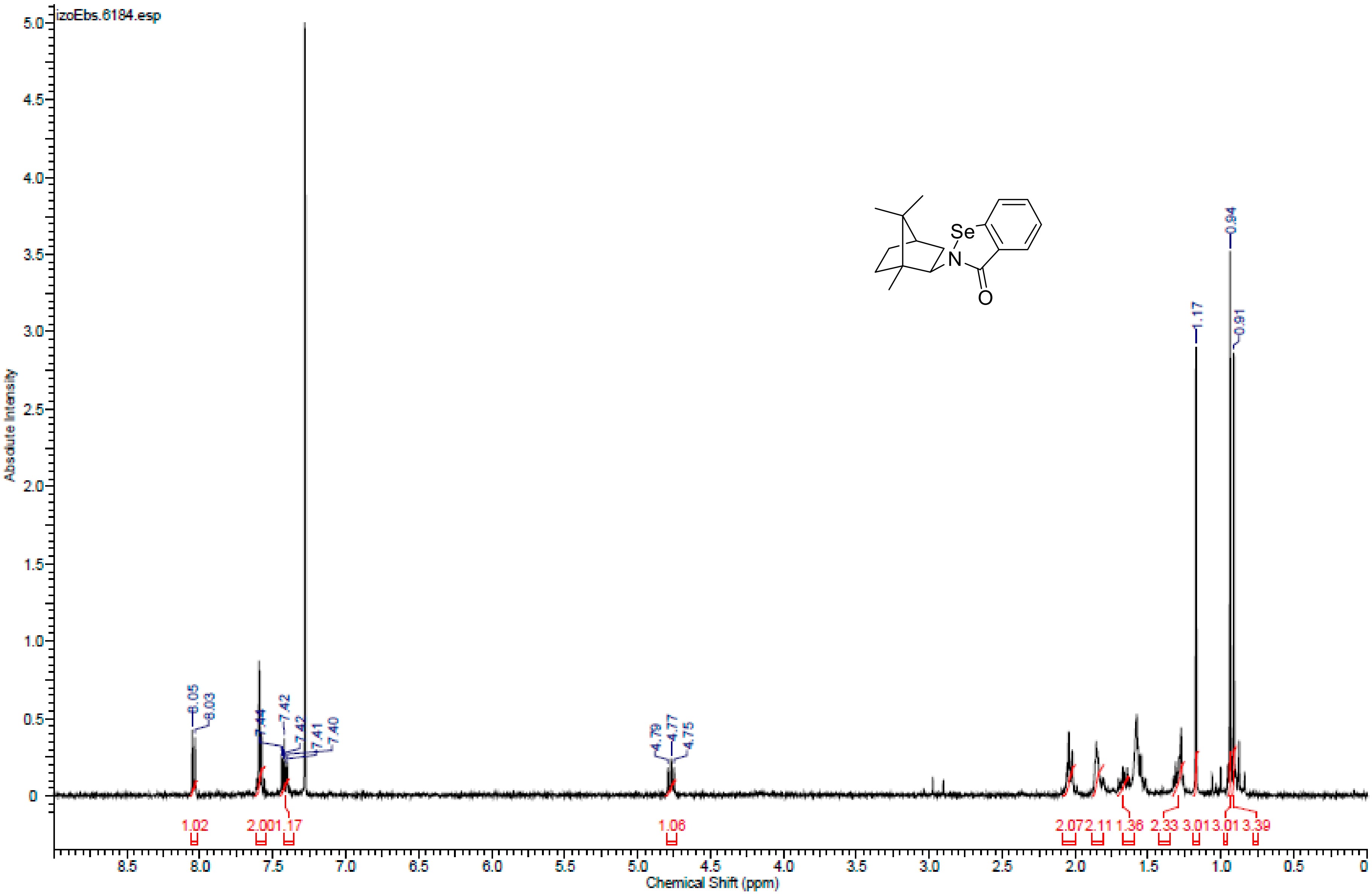

***N*-Isobornyl-1,2-benzisoselenazol-3(2*H*)-one (19)**

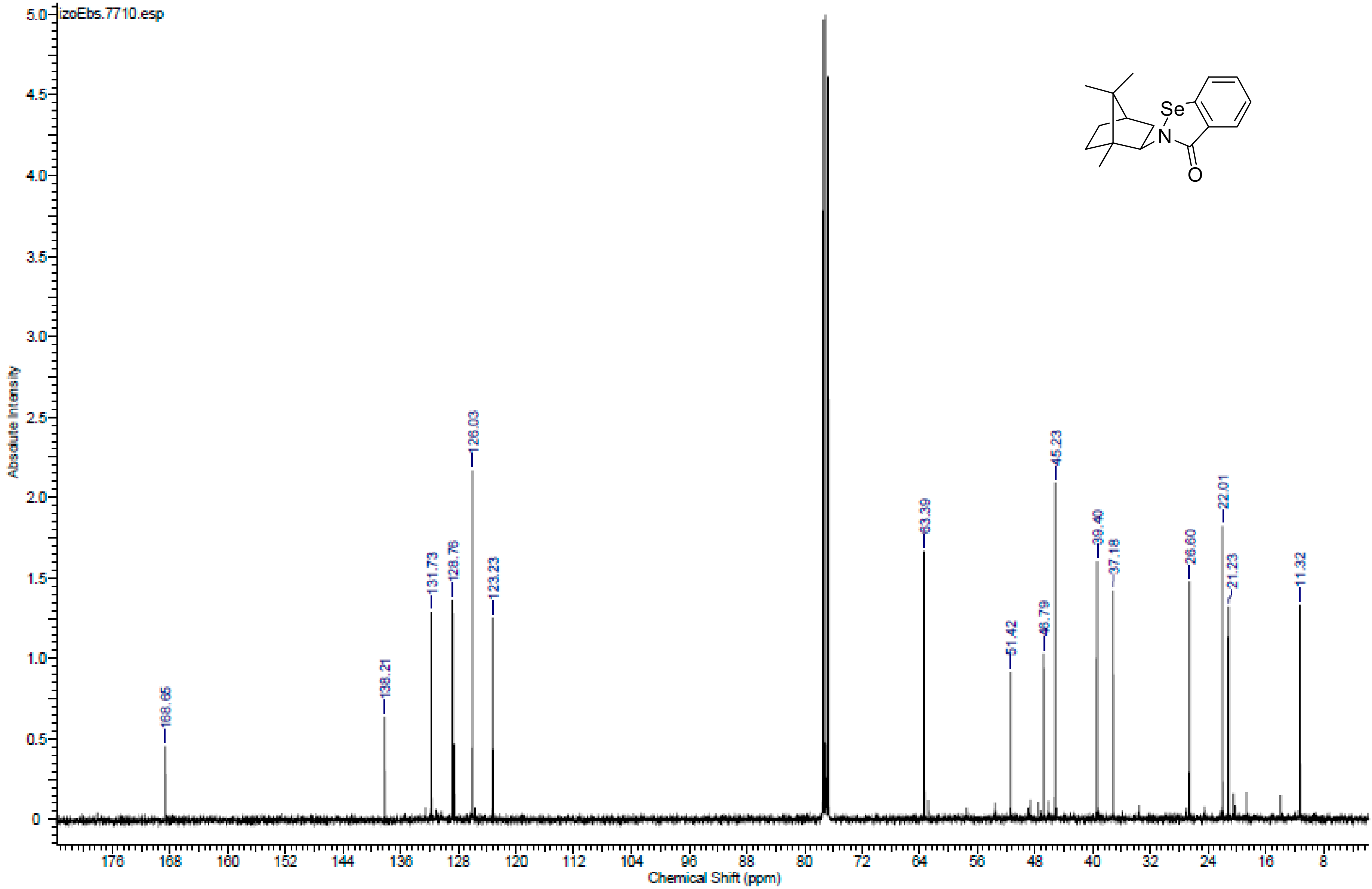

N-Isobornyl-1,2-benzisoselenazol-3(2H)-one (19)

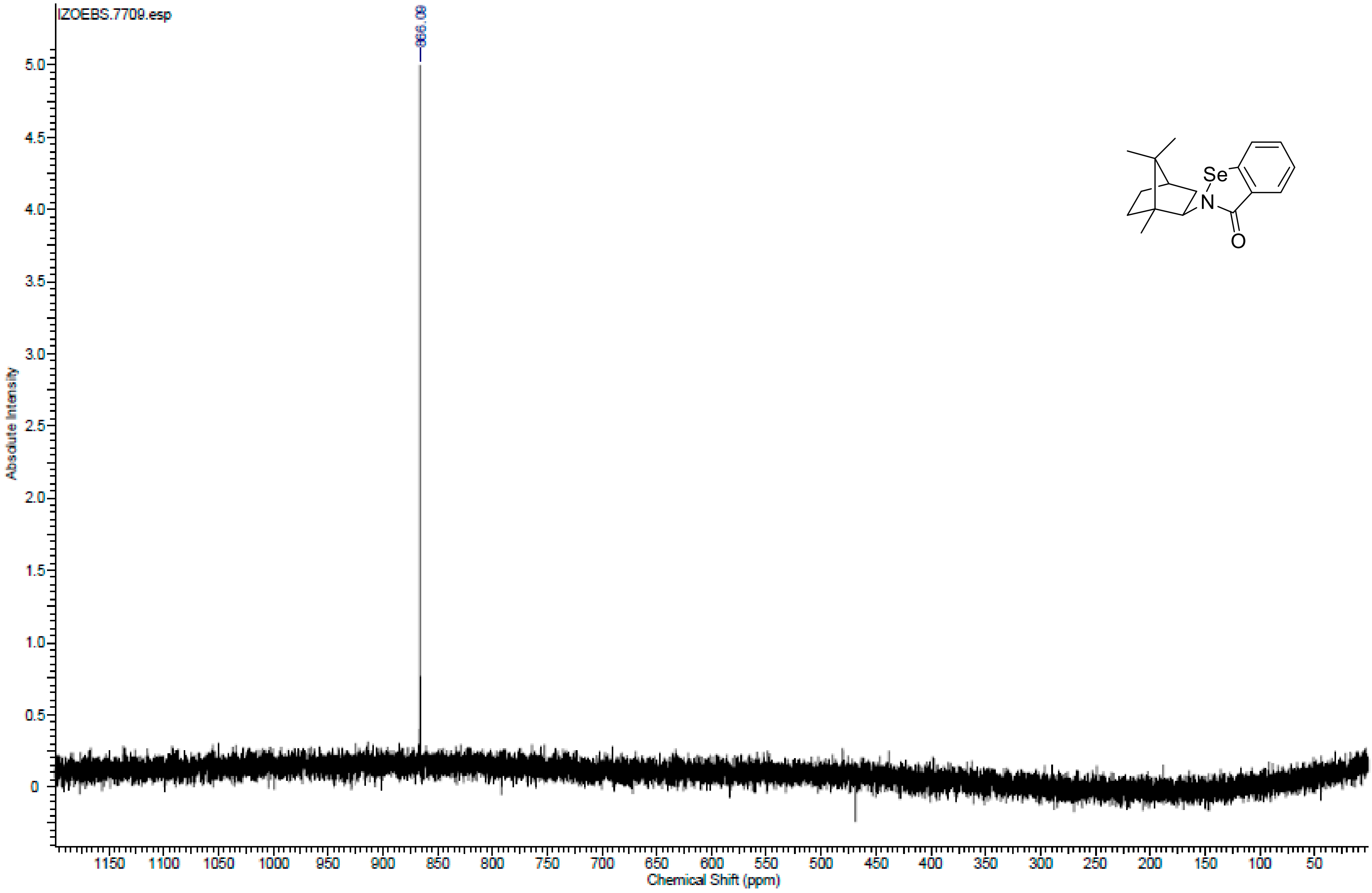

***N*-Bornyl-1,2-benzisoselenazol-3(2*H*)-one (20)**

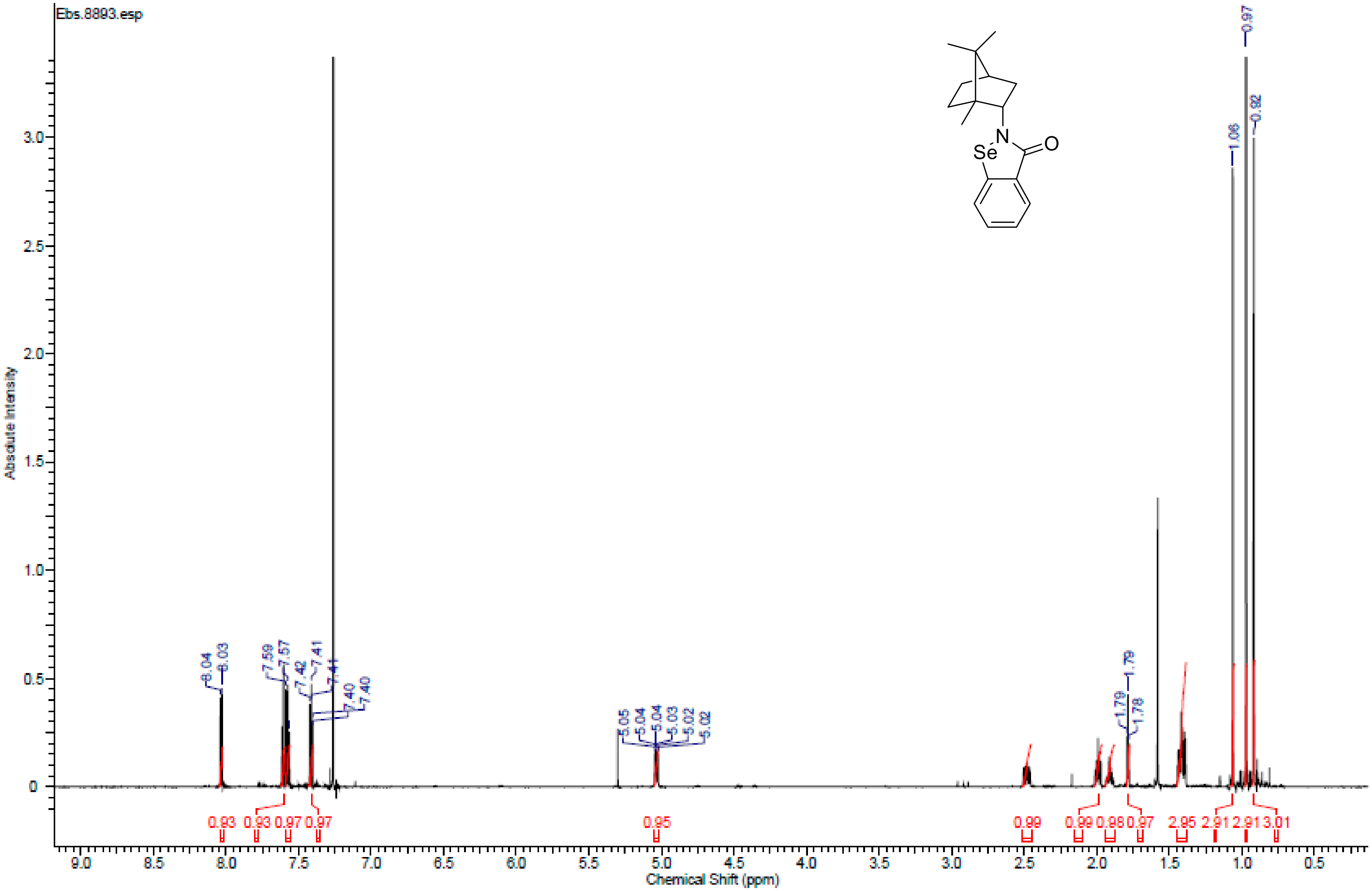

***N*-Bornyl-1,2-benzisoselenazol-3(2*H*)-one (20)**

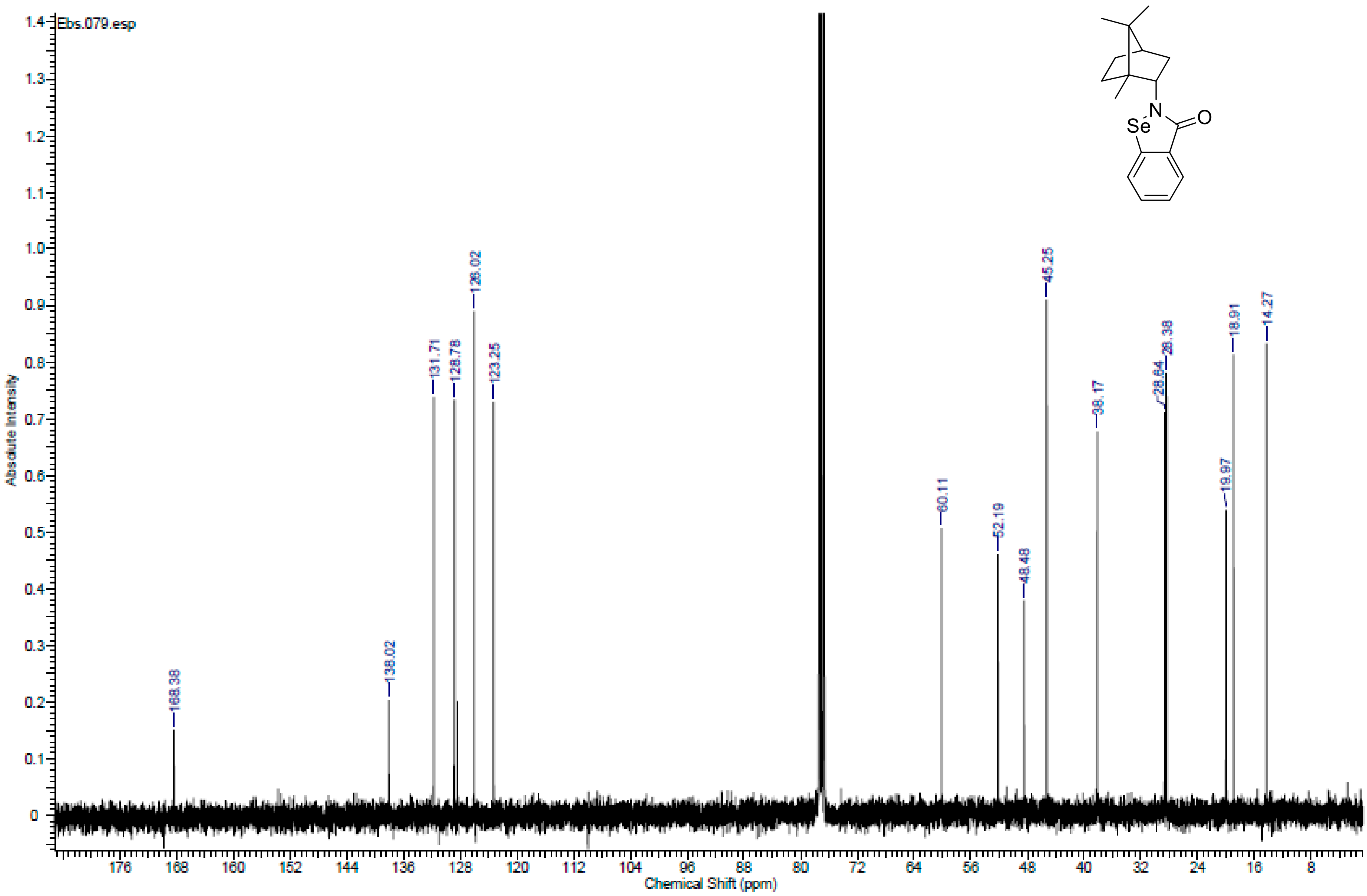

N-Bornyl-1,2-benzisoselenazol-3(2H)-one (20)

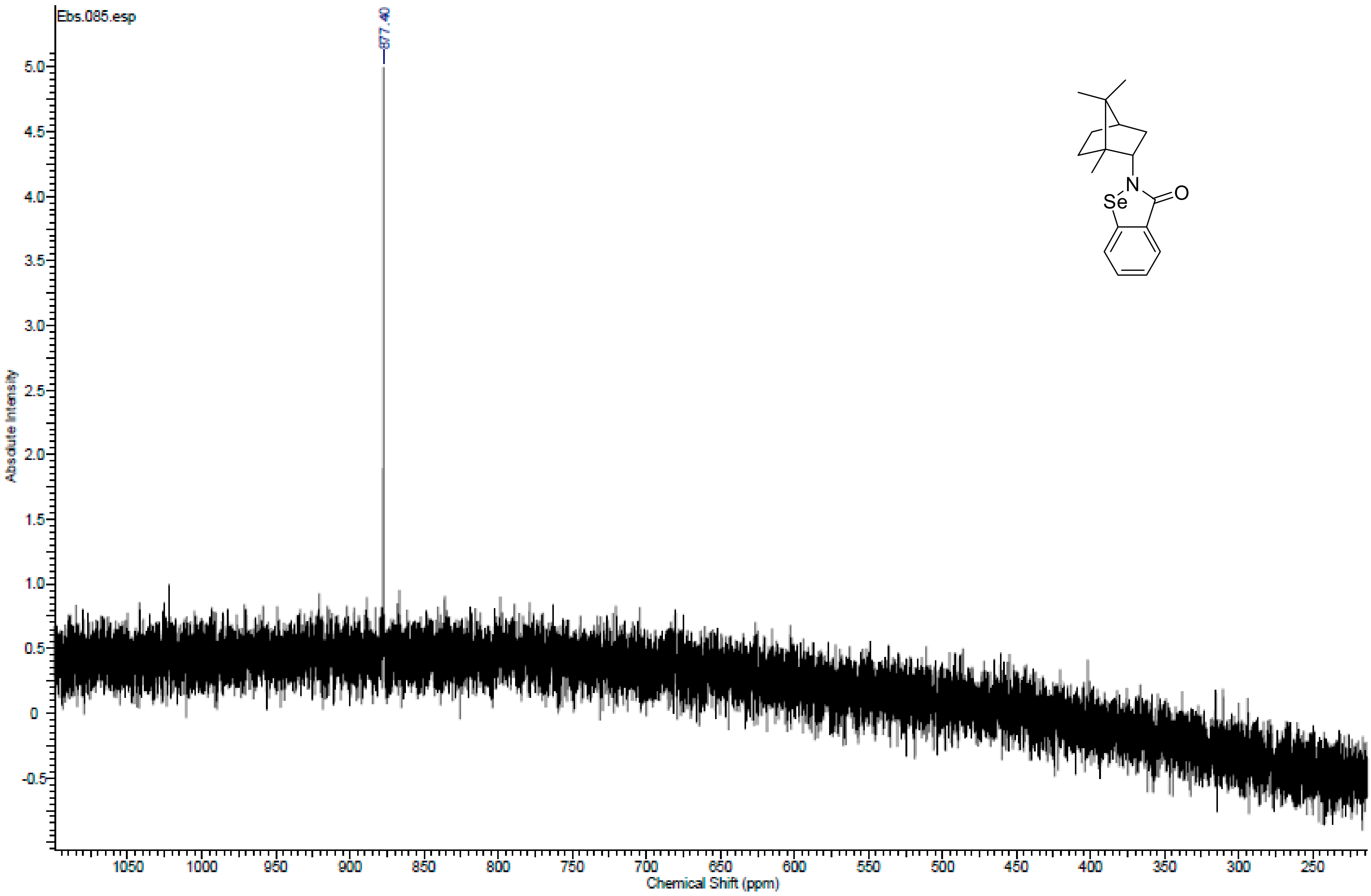

2,2-Diselenobis(benzoic acid) (6)

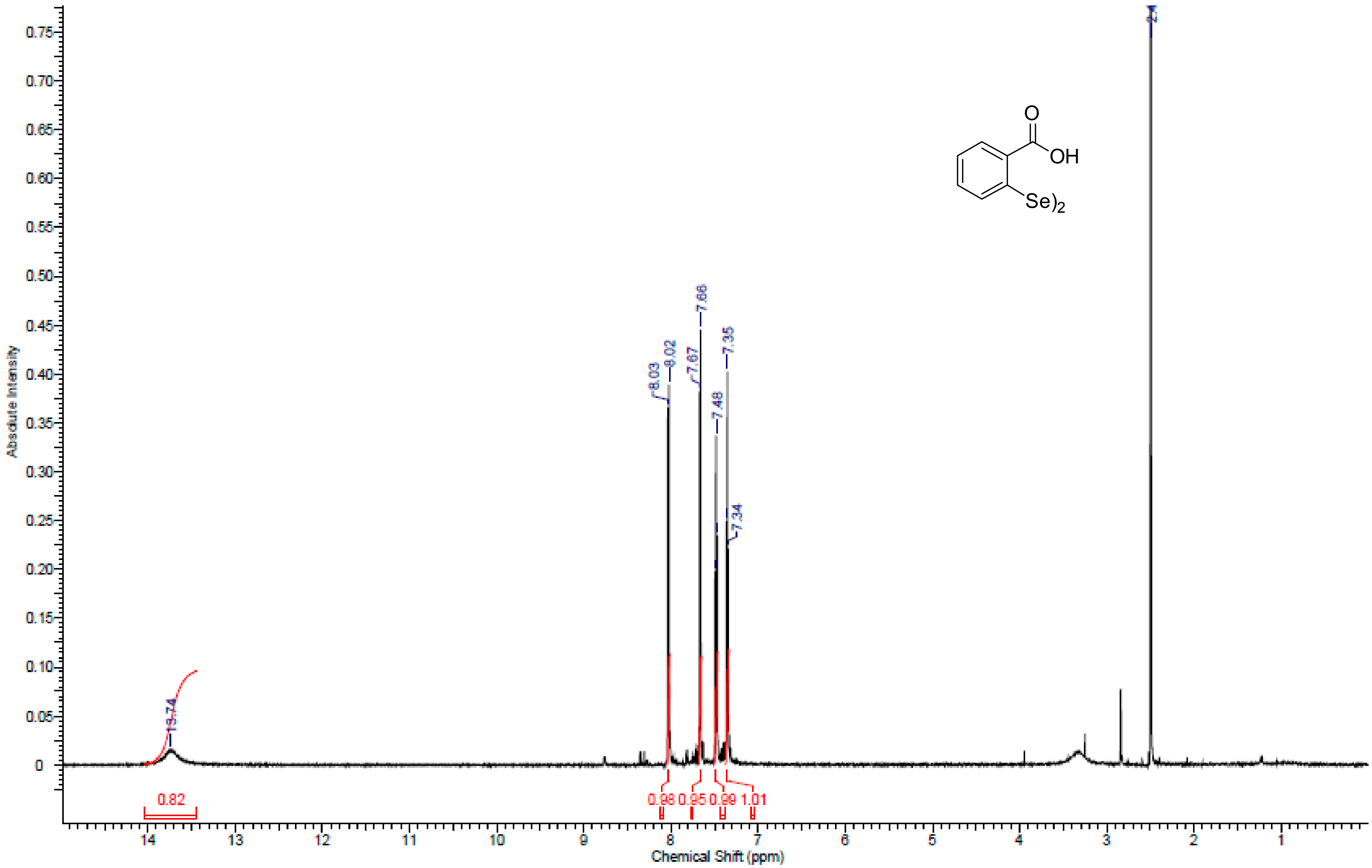

2,2-Diselenobis(benzoic acid) (6)

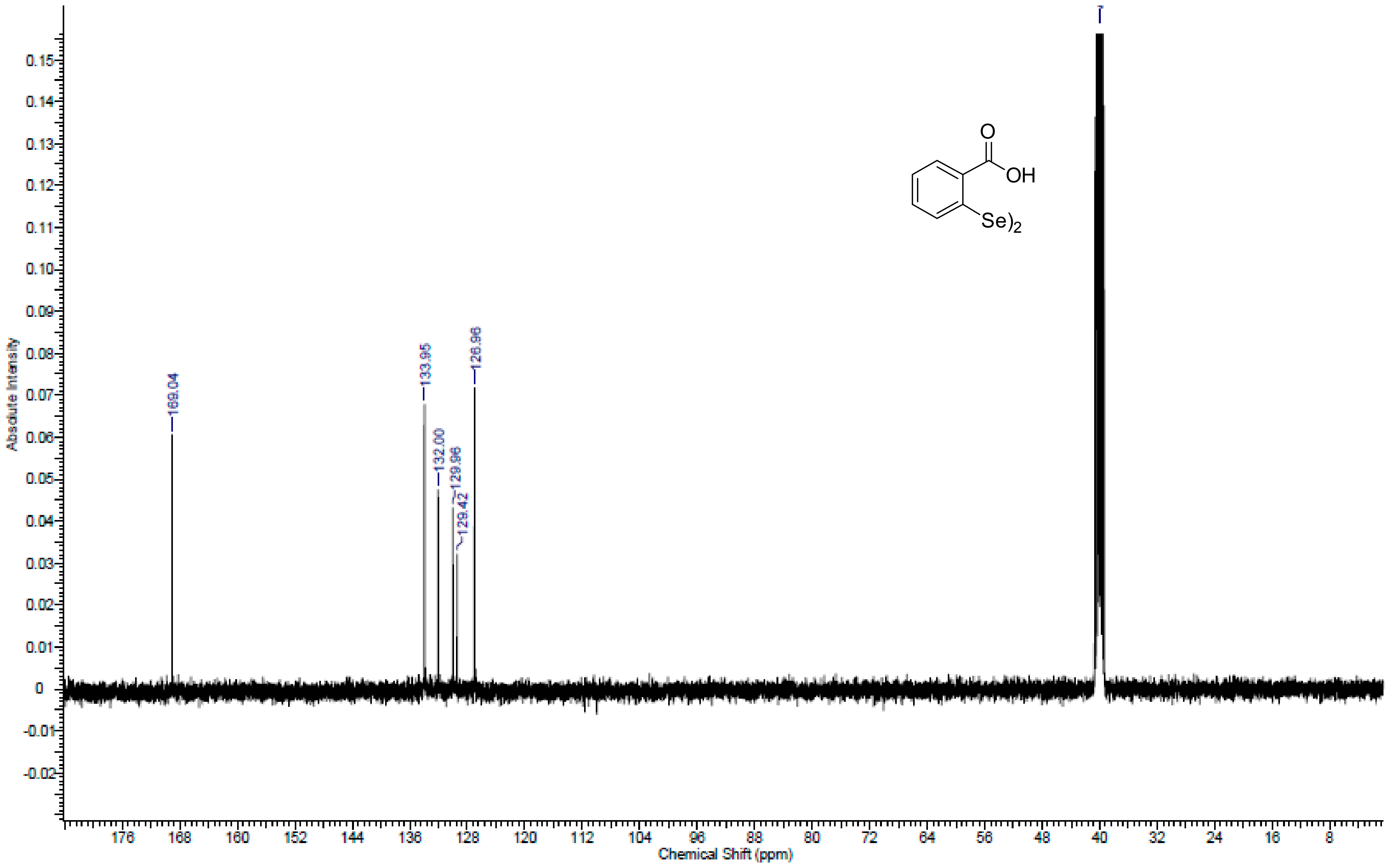

2,2-Diselenobis(benzoic acid) (6)

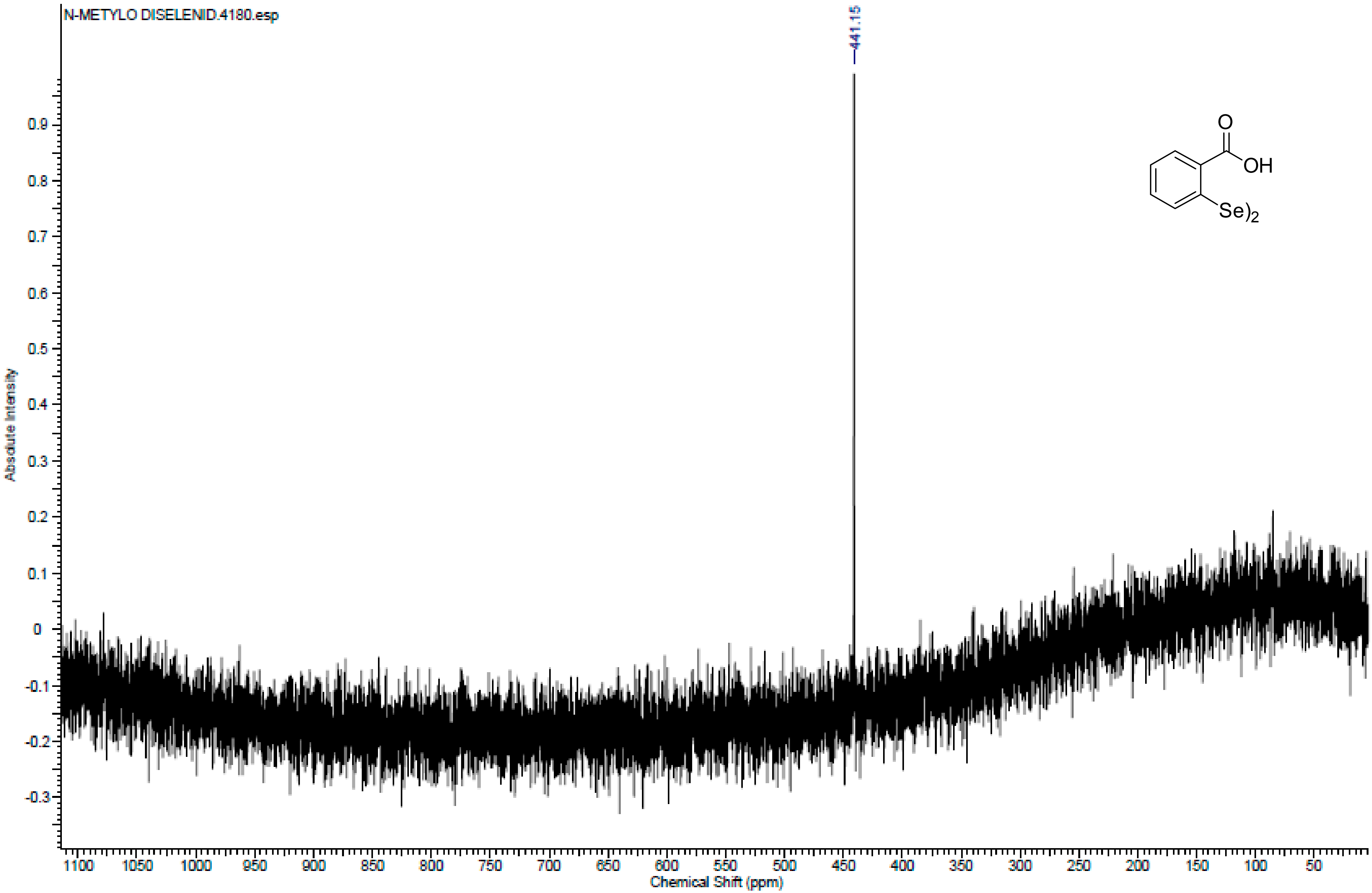

2-(Chloroseleno)benzoyl chloride (7)

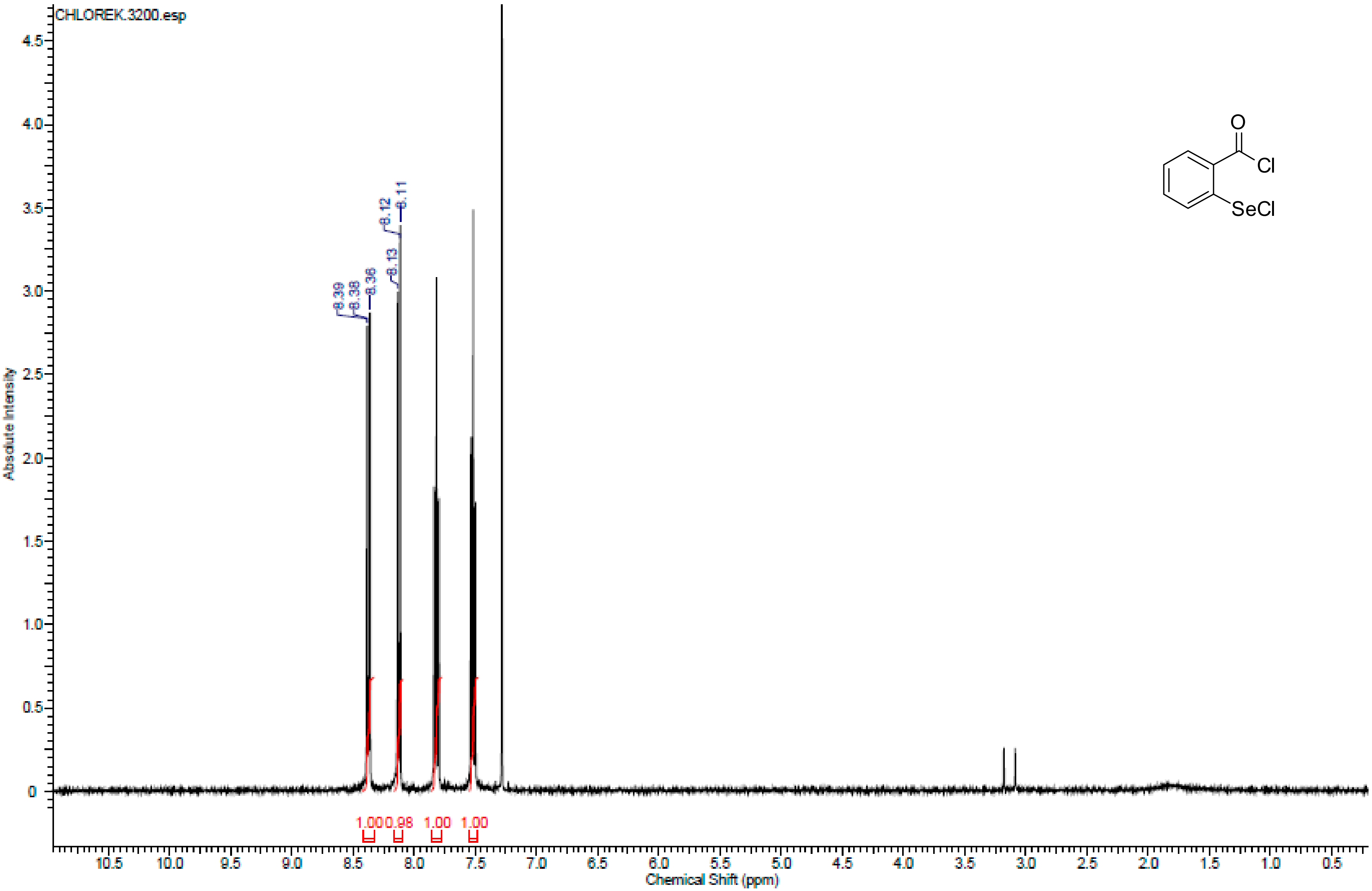

2-(Chloroseleno)benzoyl chloride (7)

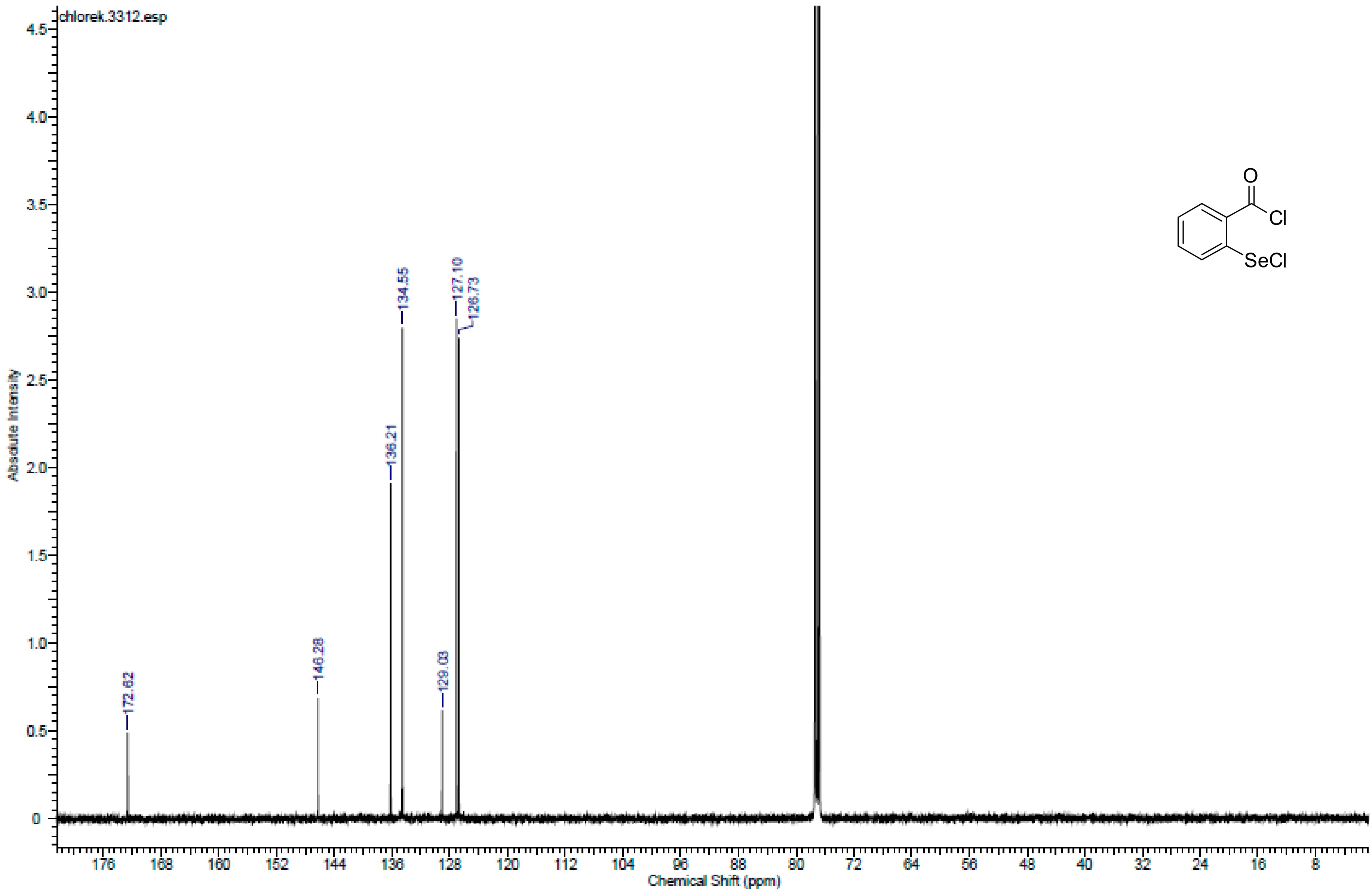

2-(Chloroseleno)benzoyl chloride (7)

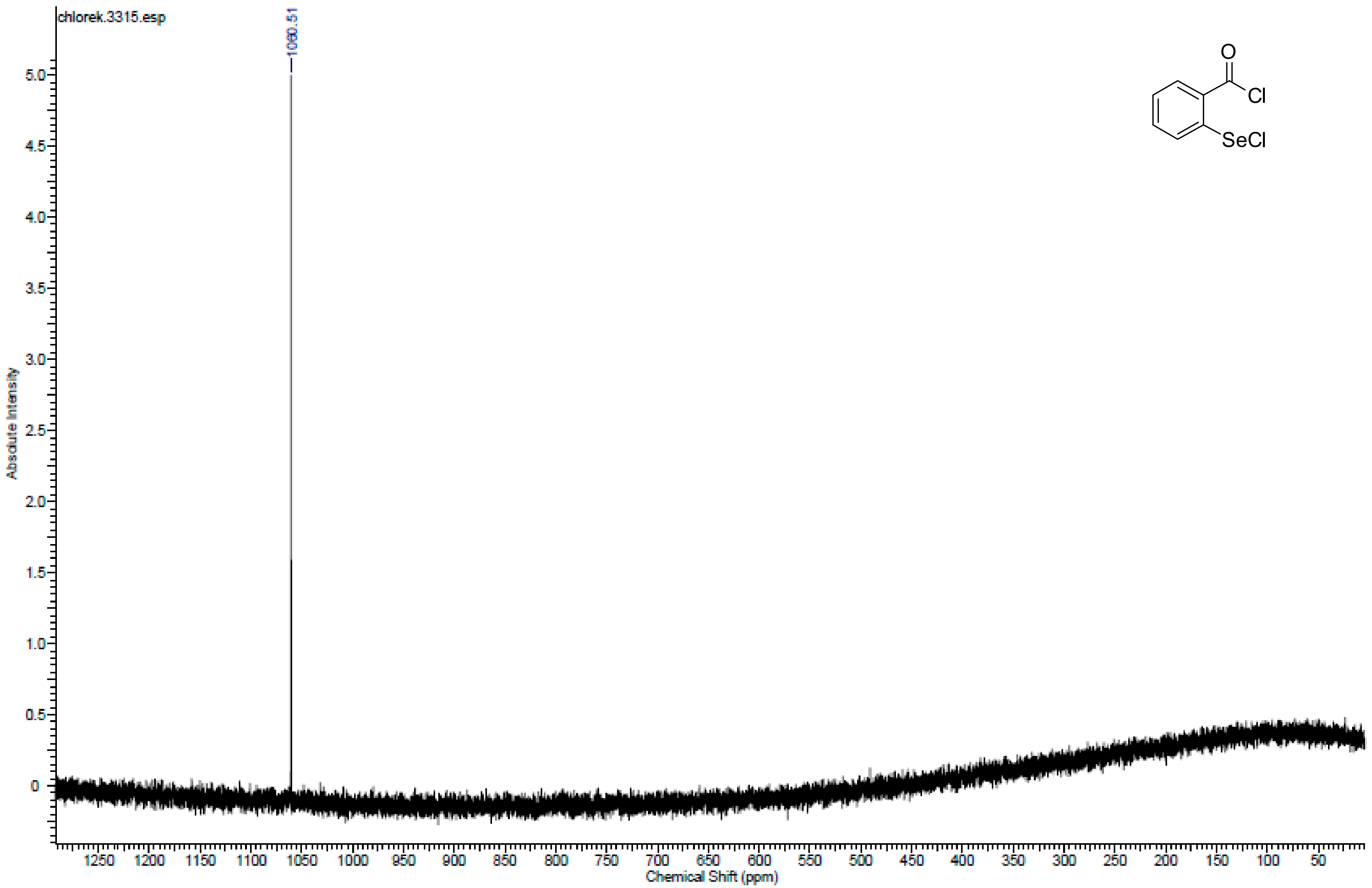

***N*-(Glycine methyl ester)-benzisoselenazol-3(2*H*)-one (21)**

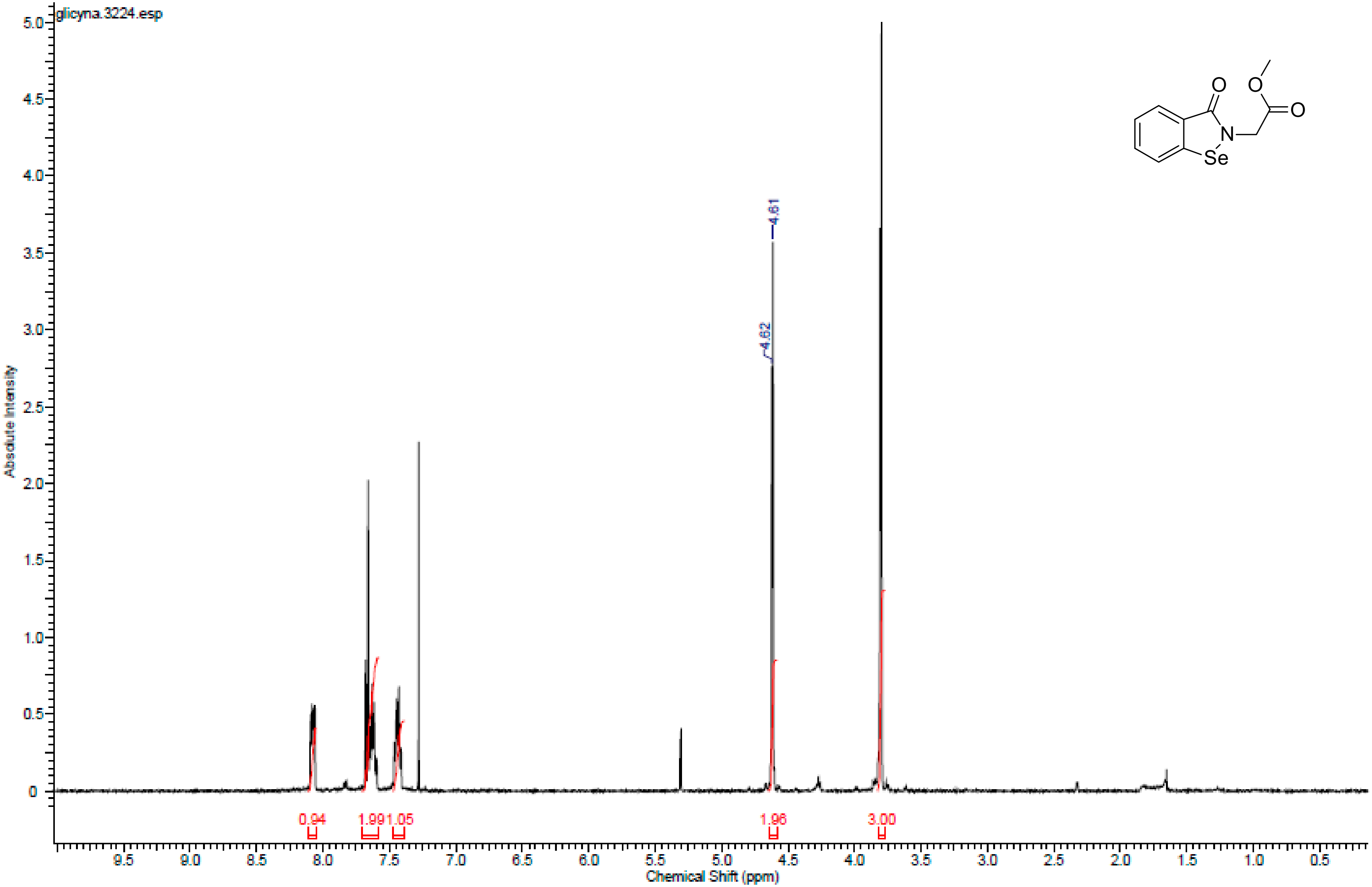

***N*-(Glycine methyl ester)-benzisoselenazol-3(2*H*)-one (21)**

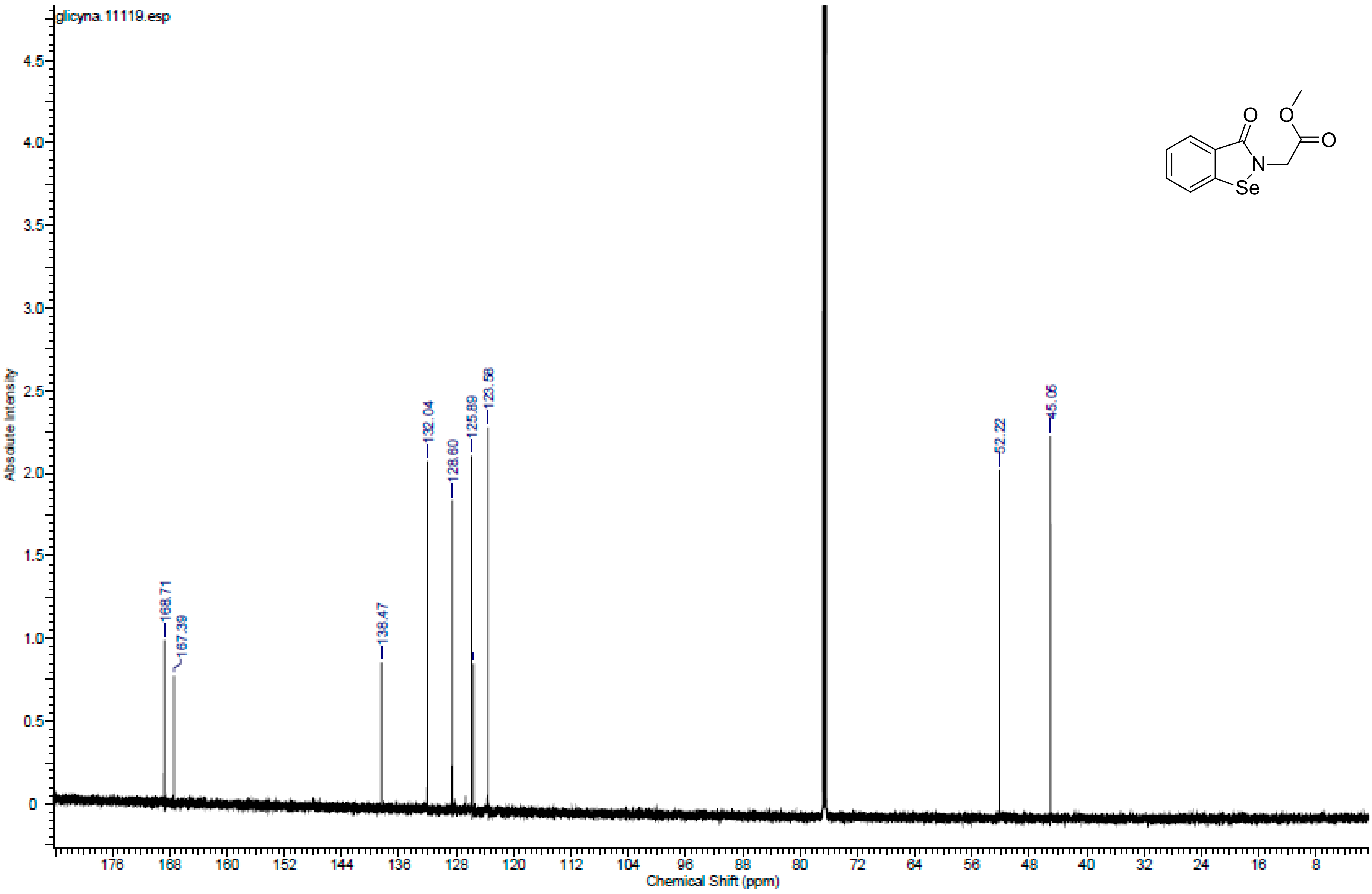

***N*-(Glycine methyl ester)-benzisoselenazol-3(2*H*)-one (21)**

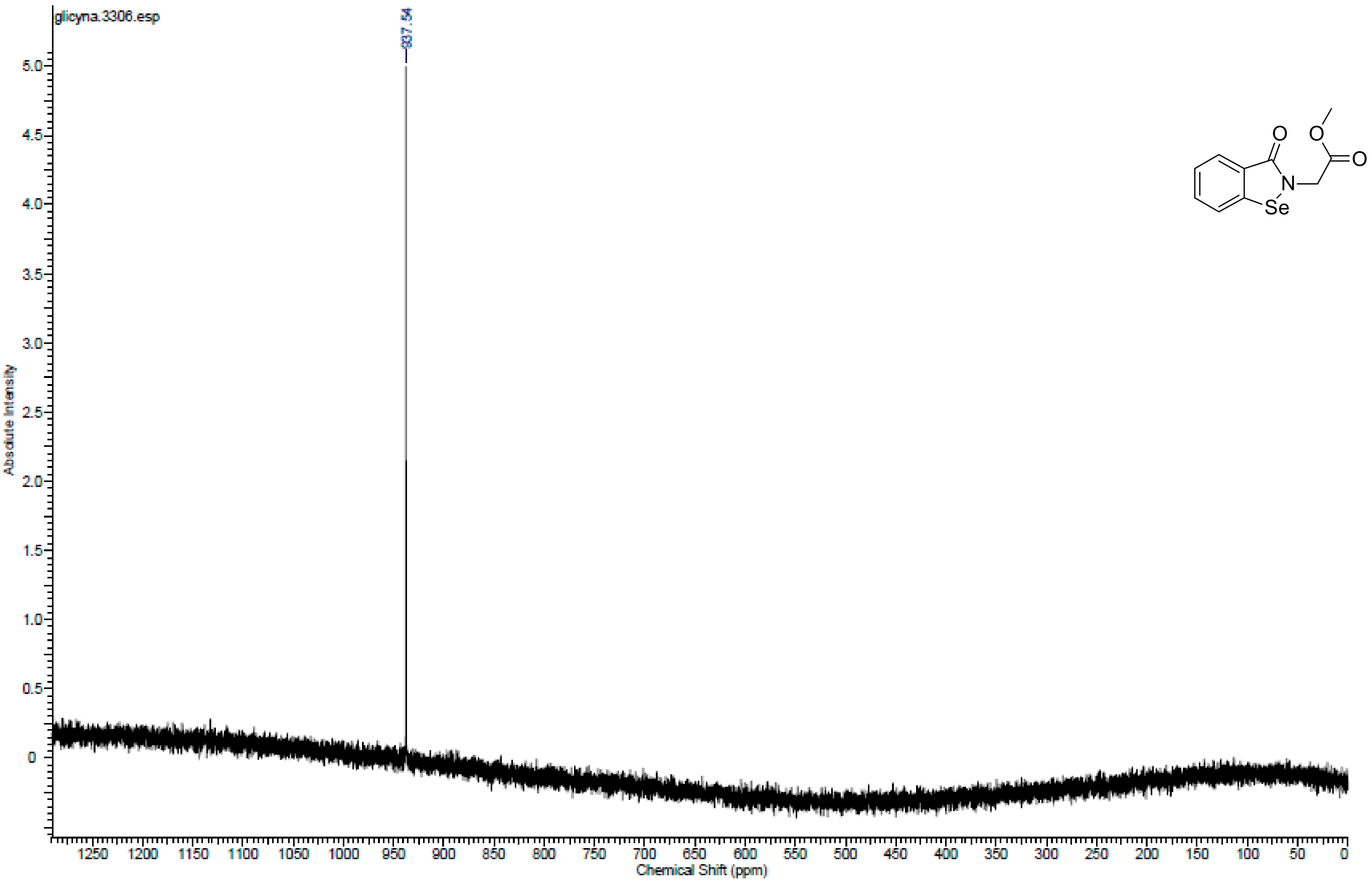

N-(Alanine methyl ester)-benzisoselenazol-3(2H)-one (22)

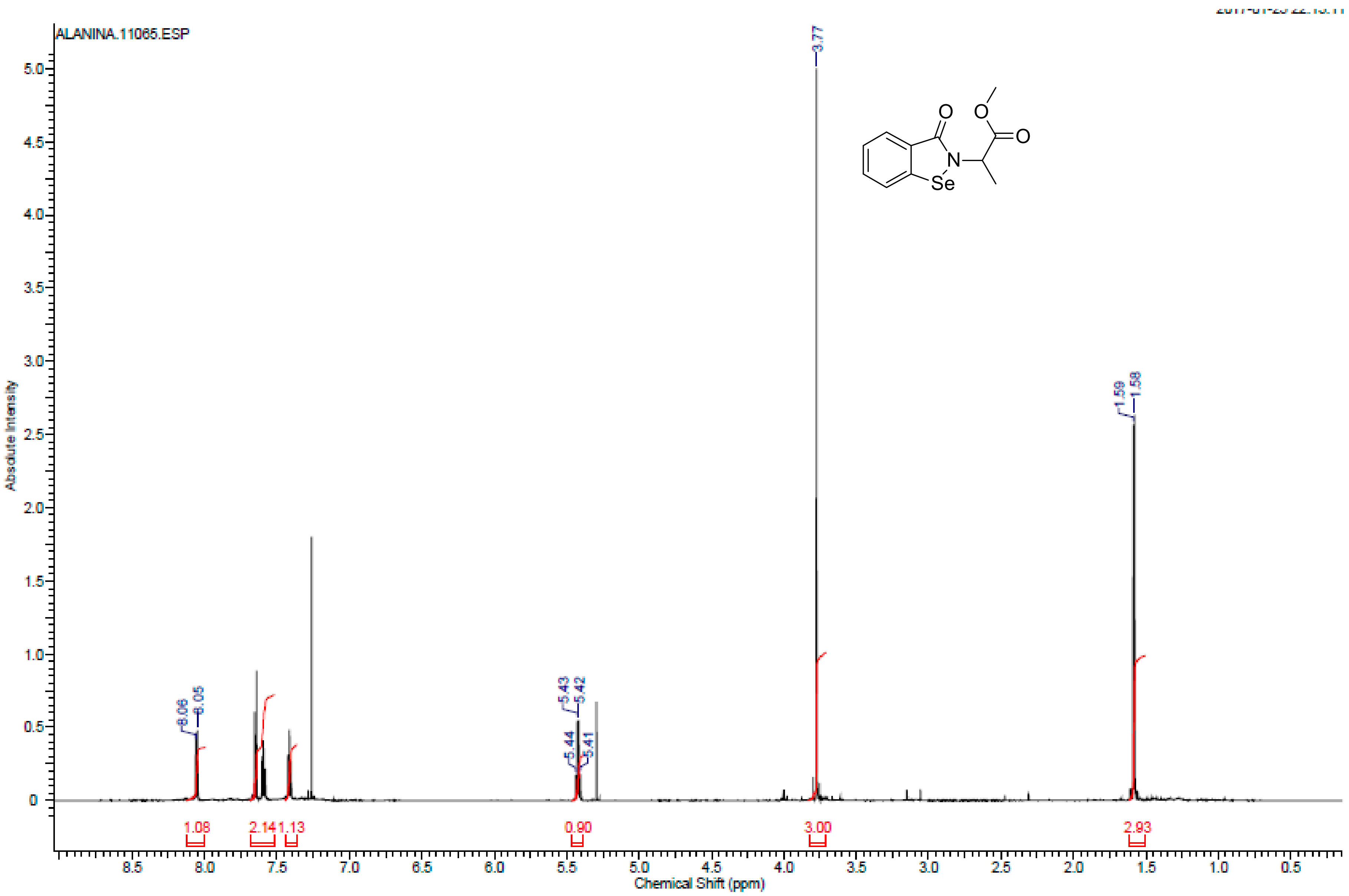

***N*-(Alanine methyl ester)-benzisoselenazol-3(2*H*)-one (22)**

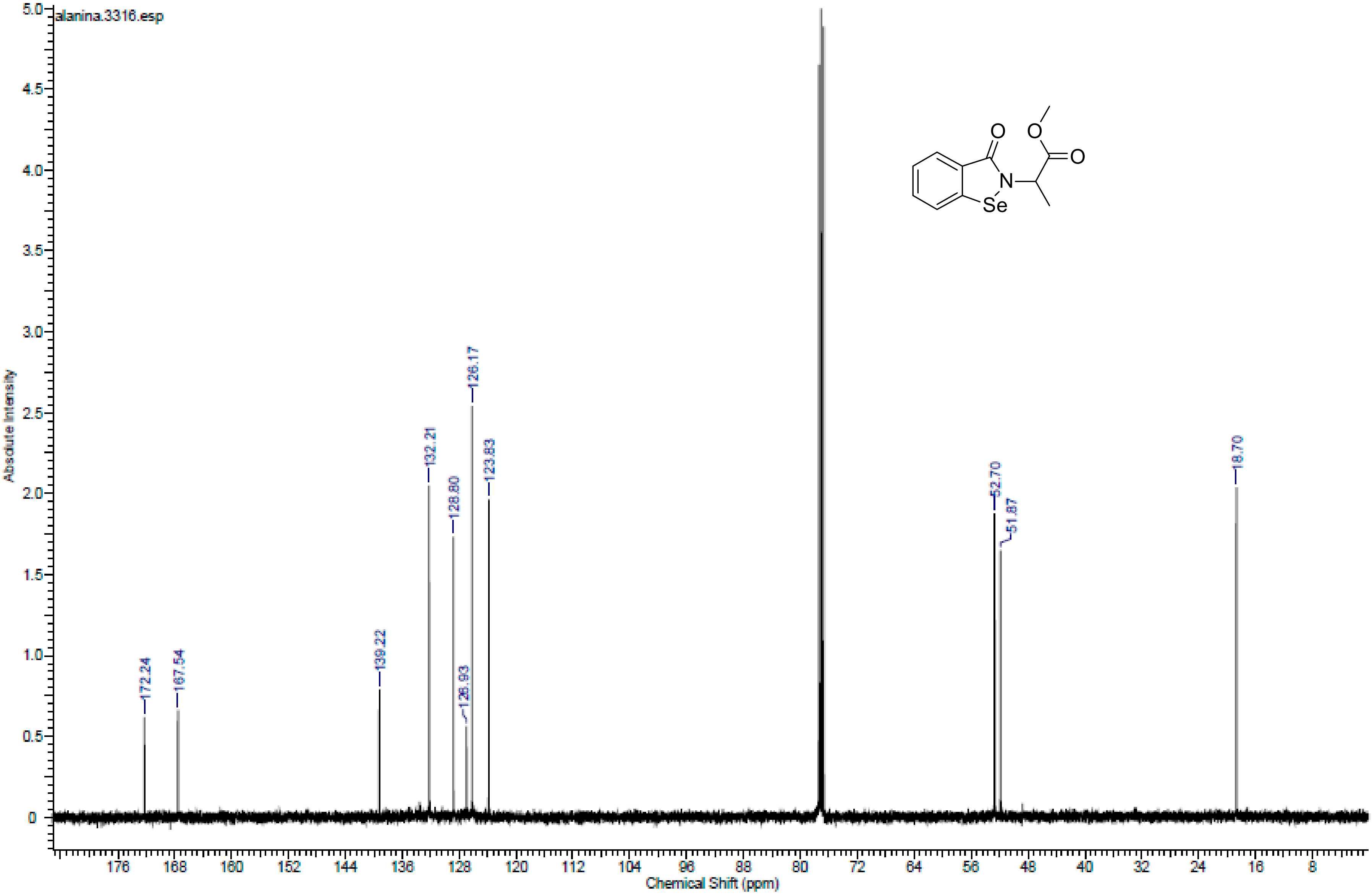

_N_-(Alanine methyl ester)-benzisoselenazol-3(2_H_)-one (22)

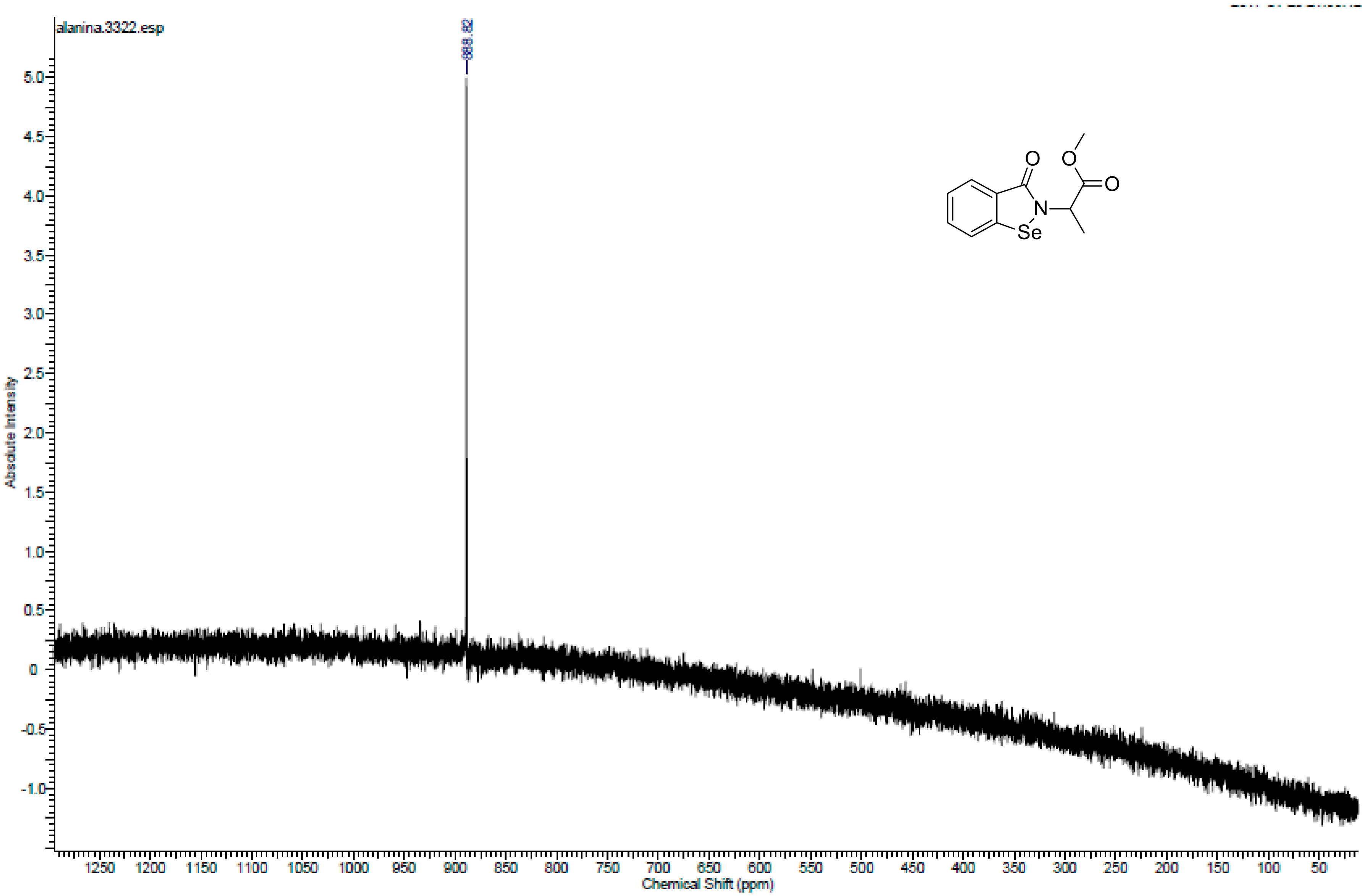

N-(Leucine methyl ester)-benzisoselenazol-3(2H)-one (23)

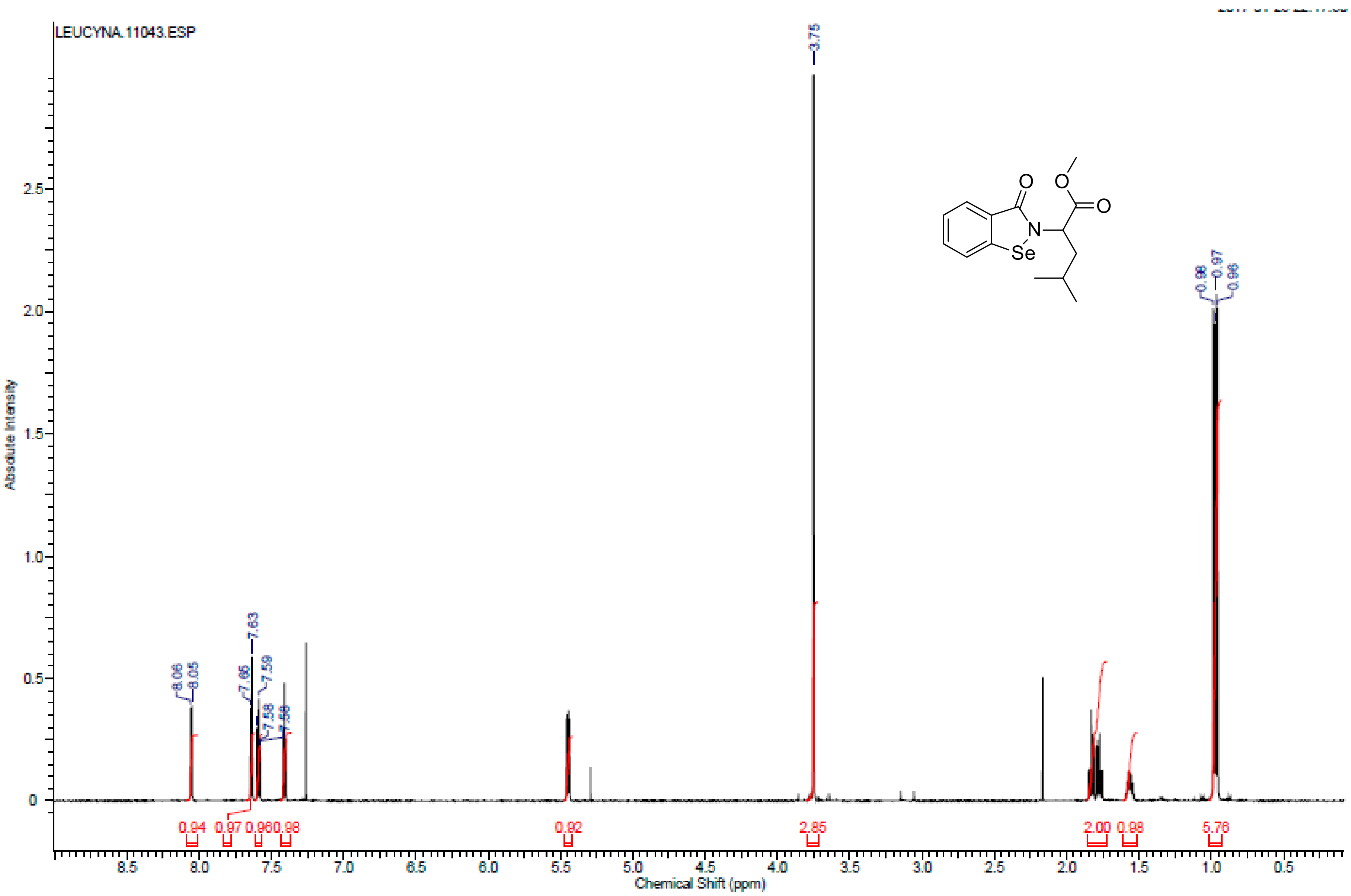

***N*-(Leucine methyl ester)-benzisoselenazol-3(2*H*)-one (23)**

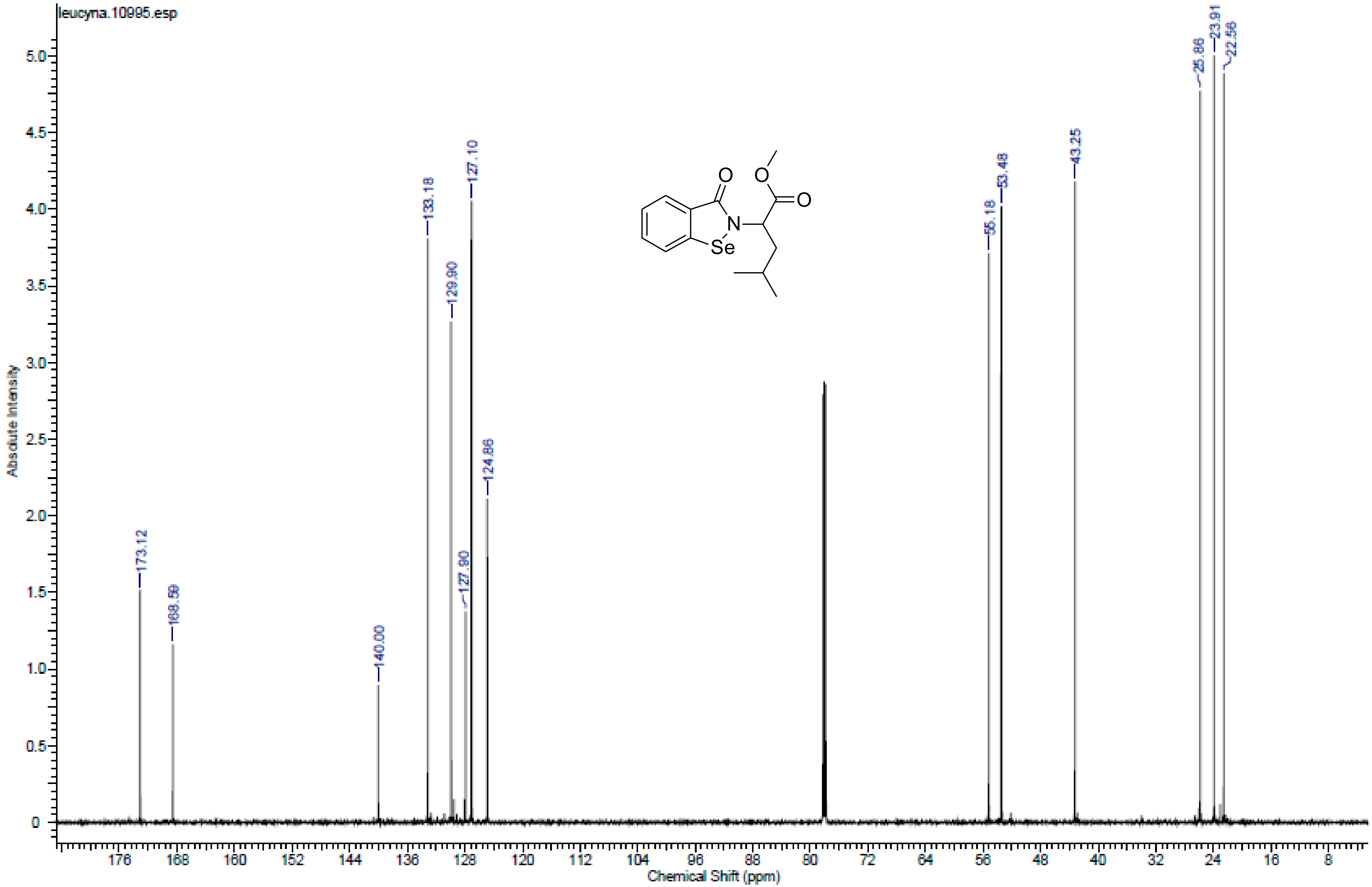

_N_-(Leucine methyl ester)-benzisoselenazol-3(2_H_)-one (23)

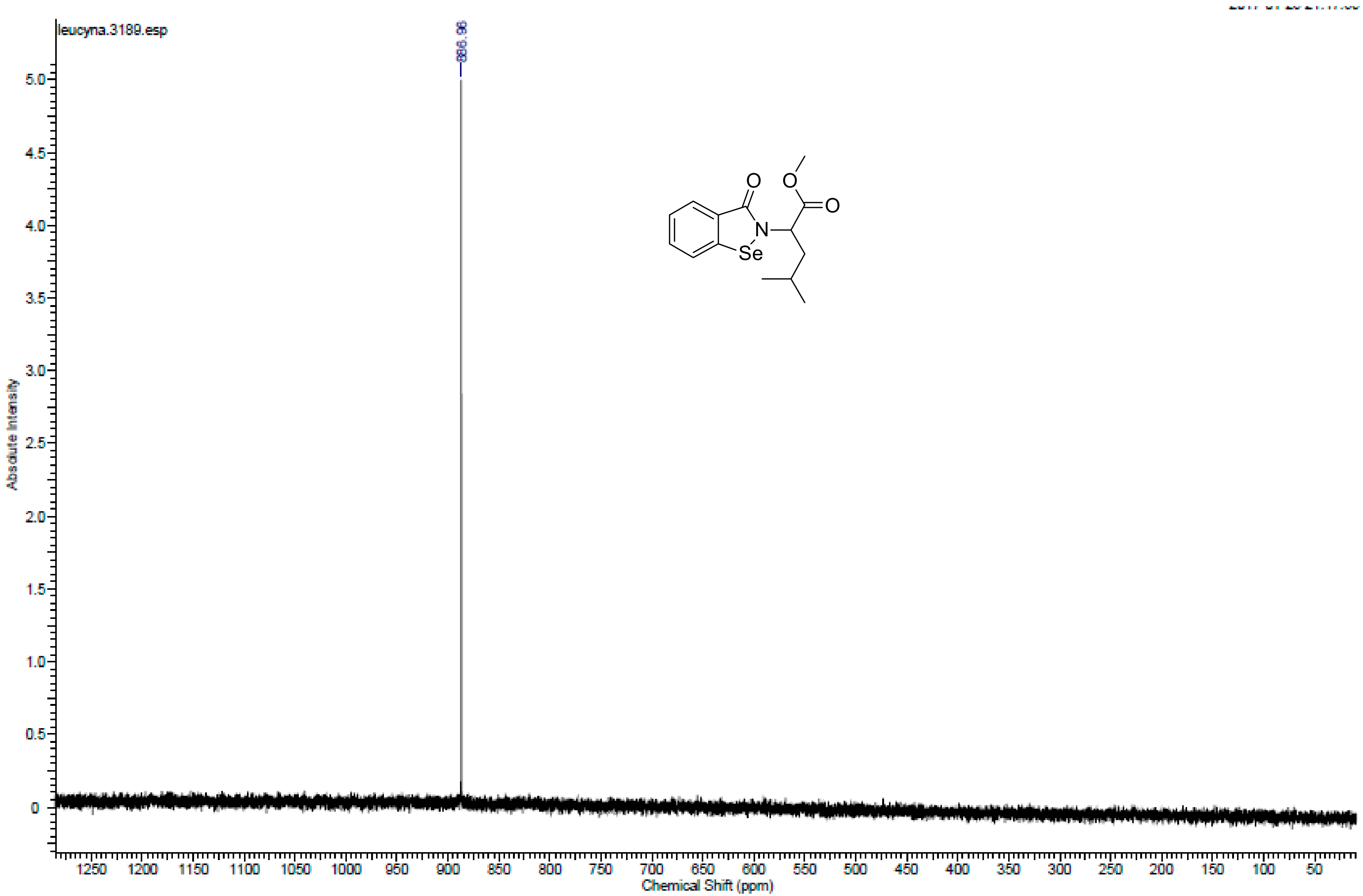

_N_-(Phenylalanine methyl ester)-benzisoselenazol-3(2_H_)-one (24)

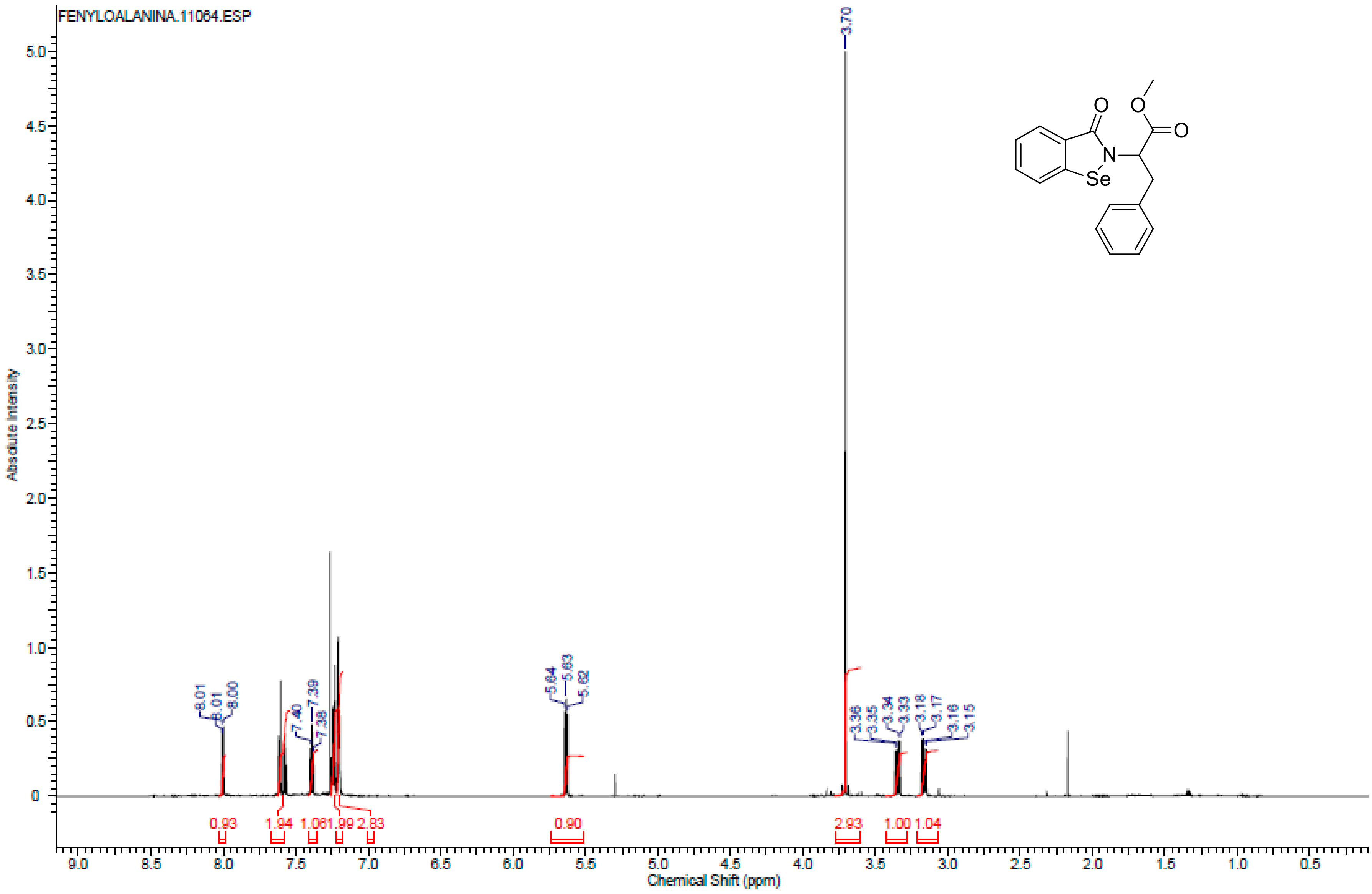

***N*-(Phenylalanine methyl ester)-benzisoselenazol-3(2*H*)-one (24)**

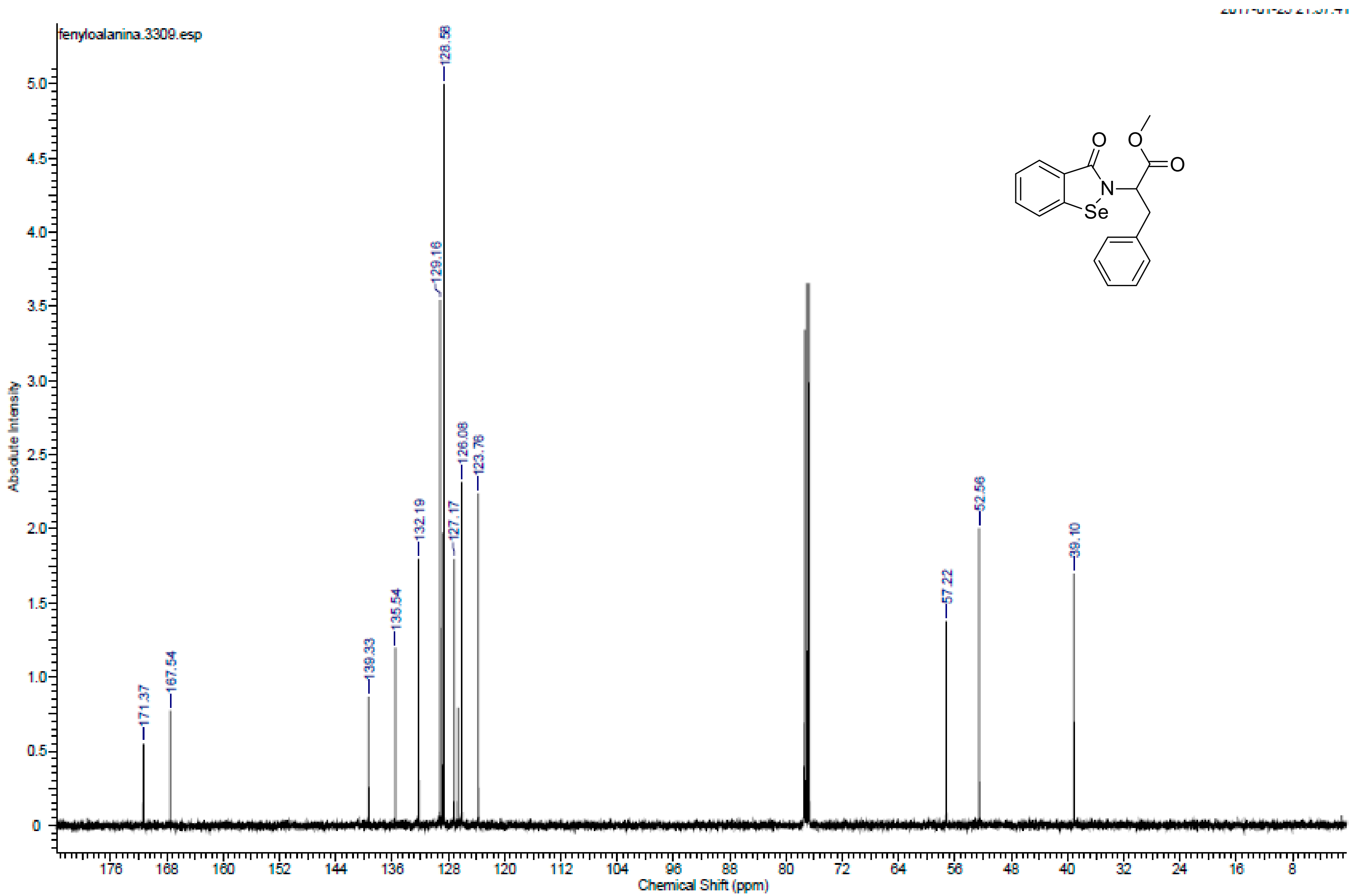

N-(Phenylalanine methyl ester)-benzisoselenazol-3(2H)-one (24)

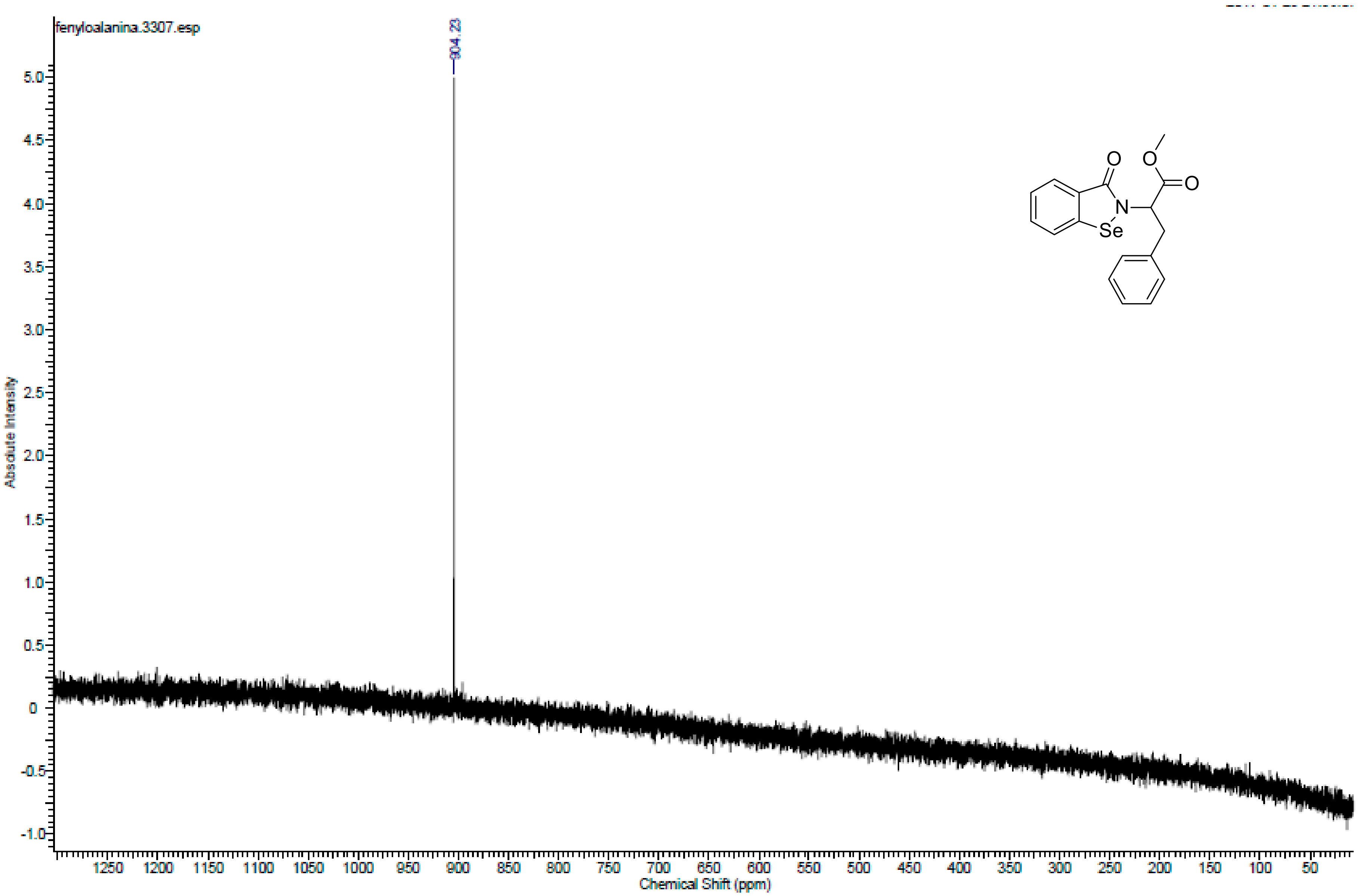

N-(Alanine carboxyl acid)-benzisoselenazol-3(2H)-one (25)

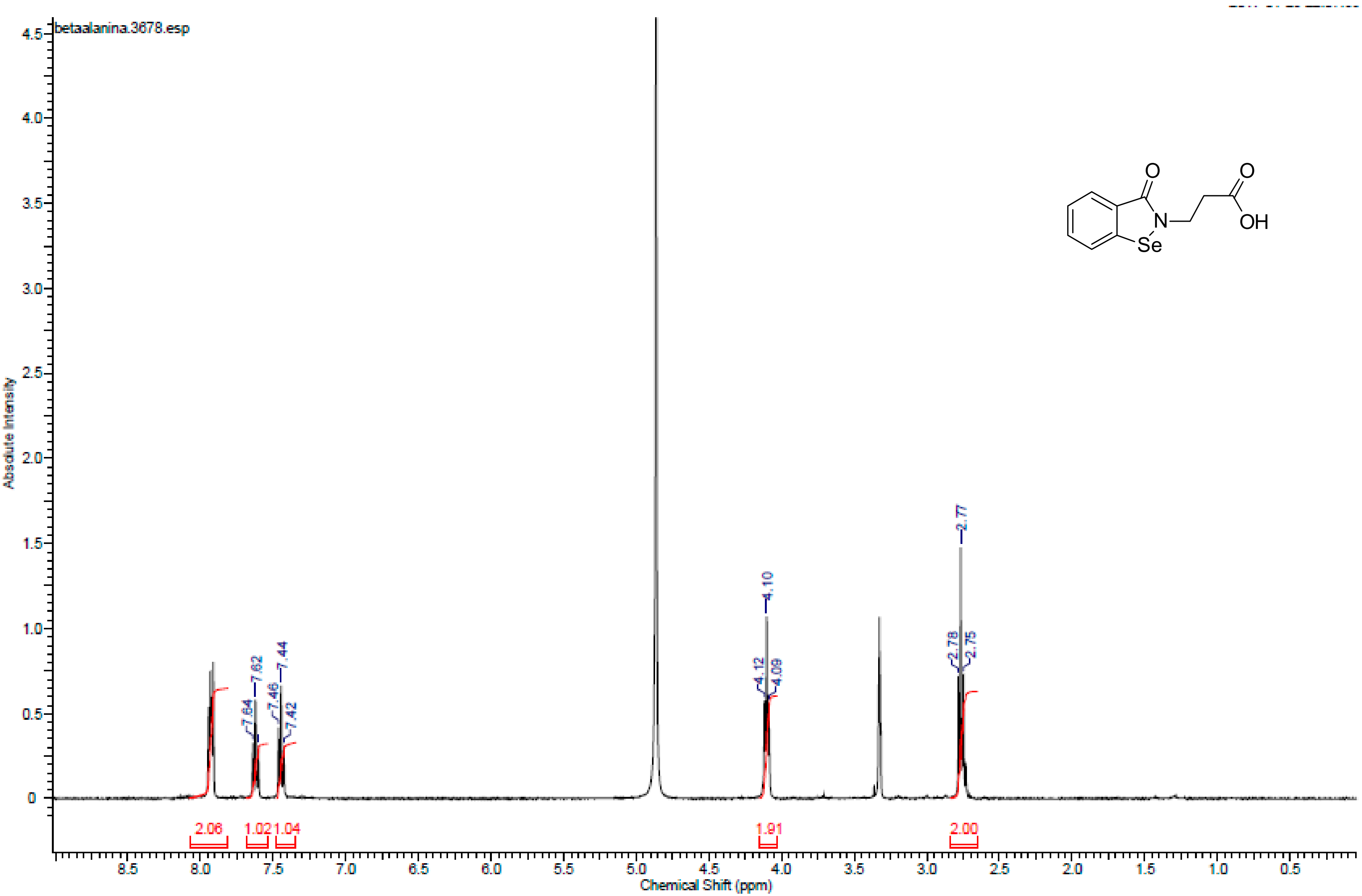

***N*-(Alanine carboxyl acid)-benzisoselenazol-3(2*H*)-one (25)**

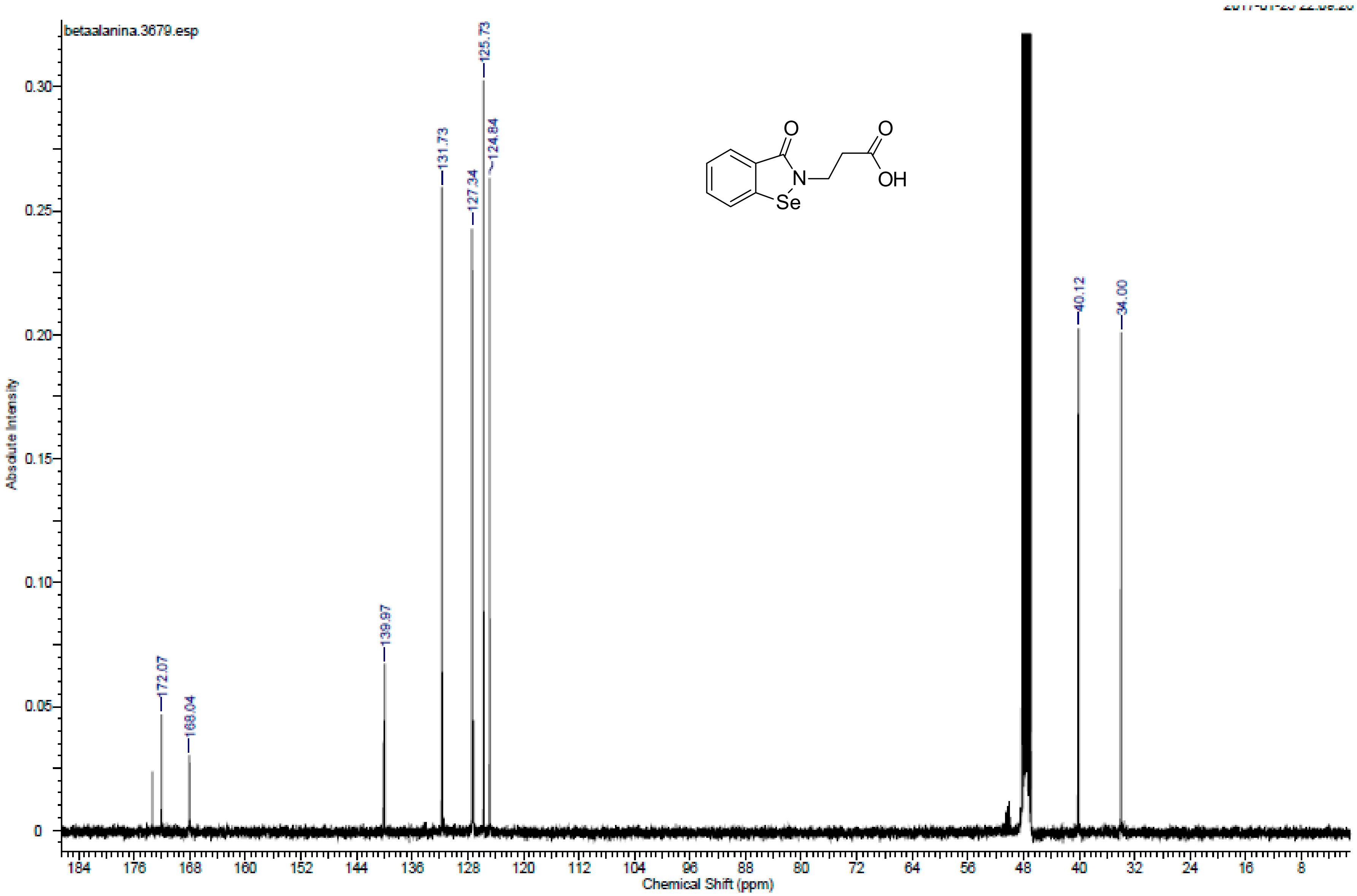

***N*-(Alanine carboxyl acid)-benzisoselenazol-3(2*H*)-one (25)**

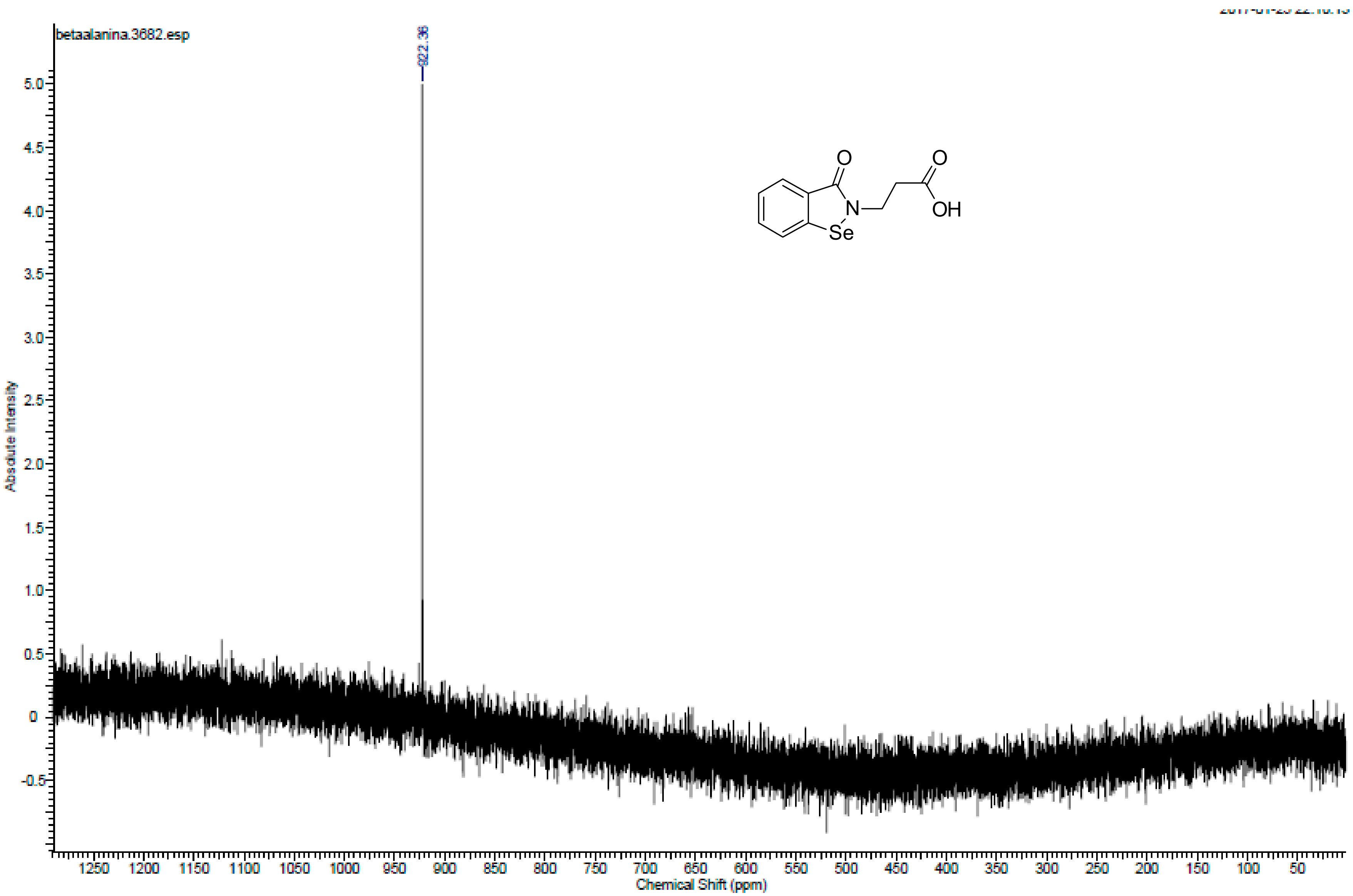

VI. Crystallographic data

Table 1. Crystal data and structure refinement for *N*-bornyl-1,2-benzisoselenazol-3(2*H*)-one **20** (**e654a**).

Identification code	e654a
Empirical formula	C17 H21 N O Se
Formula weight	334.31
Temperature; K	293(2)
Wavelength; Å	0.71073
Crystal system, space group	Orthorhombic, P2(1)2(1)2(1)
Unit cell dimensions; Å	a = 10.7228(10)
	b = 11.5034(9)
	c = 12.0645(10)
Volume; $Å^3$	1488.1(2)
Z, Calculated density; Mg/m^3	4, 1.492
Absorption coefficient; mm^{-1}	2.519
F(000)	688
Crystal size; mm	0.49 x 0.16 x 0.13
Theta range for data collection	2.45 to 28.03 deg.
Limiting indices	-13<=h<=13, -14<=k<=15, -15<=l<=13
Reflections collected / unique	9666 / 3266 [R(int) = 0.0257]
Completeness to theta	26.00 100.0 %
Absorption correction	Analytical
Max. and min. transmission	0.7371 and 0.4099
Refinement method	Full-matrix least-squares on F^2
Data / restraints / parameters	3266 / 0 / 181
Goodness-of-fit on F^2	0.995
Final R indices [I>2sigma(I)]	R1 = 0.0205, wR2 = 0.0446
R indices (all data)	R1 = 0.0248, wR2 = 0.0458
Absolute structure parameter	-0.008(4)
Largest diff. peak and hole; $e.A^{-3}$	0.218 and -0.320

Table 2. Selected bond lengths [Å] and angles [º] for *N*-bornyl-1,2-benzisoselenazol-3(2*H*)-one **20** e654a.

C1-C10	1.515(4)
C1-C6	1.533(4)
C1-C7	1.553(4)
C1-C2	1.558(4)
C2-N1	1.461(3)

C2-C3	1.543(4)
C3-C4	1.530(4)
C4-C5	1.522(4)
C4-C7	1.537(4)
C5-C6	1.552(4)
C7-C9	1.530(4)
C7-C8	1.533(4)
Se1-N1	1.883(2)
Se1-C17	1.887(2)
N1-C11	1.358(3)
C11-O1	1.235(3)
C11-C12	1.472(3)
C12-C17	1.380(4)
C12-C13	1.389(4)
C13-C14	1.376(4)
C14-C15	1.393(4)
C15-C16	1.376(4)
C16-C17	1.388(3)
C10-C1-C6	113.7(2)
C10-C1-C7	117.7(2)
C6-C1-C7	102.1(2)
C10-C1-C2	114.3(2)
C6-C1-C2	107.5(2)
C7-C1-C2	100.0(2)
N1-C2-C3	115.6(2)
N1-C2-C1	116.7(2)
C3-C2-C1	103.1(2)
C4-C3-C2	103.4(2)
C5-C4-C3	107.3(2)

C5-C4-C7	102.9(2)
C3-C4-C7	102.5(2)
C4-C5-C6	102.7(2)
C1-C6-C5	103.9(2)
C9-C7-C8	106.3(3)
C9-C7-C4	114.6(3)
C8-C7-C4	113.4(3)
C9-C7-C1	113.8(3)
C8-C7-C1	115.2(2)
C4-C7-C1	93.6(2)
N1-Se1-C17	85.89(10)
C11-N1-C2	121.1(2)
C11-N1-Se1	115.29(16)
C2-N1-Se1	123.35(16)
O1-C11-N1	123.3(2)
O1-C11-C12	125.4(2)
N1-C11-C12	111.3(2)
C17-C12-C13	120.0(2)
C17-C12-C11	116.0(2)
C13-C12-C11	124.0(2)
C14-C13-C12	119.7(3)
C13-C14-C15	119.5(3)
C16-C15-C14	121.6(3)
C15-C16-C17	118.1(3)
C12-C17-C16	121.1(2)
C12-C17-Se1	111.54(19)
